# Clinical Review, and Hospital Reports

**Published:** 1836-04-01

**Authors:** 


### Clinical Review.

### Guy's Hospital.

On Diseased Conditions of the Cerebral Arteries. By Dr. Bright.

Guy's Hospital has followed the example of its neighbour St. Thomas, and commenced a series of Hospital Reports, which bid fair to benefit the profession at large, while adding to the reputation of the medical officers themselves. The publication is under the care of Messrs. Barlow and Babington, and no reporters' names are given, as in the contemporary work from the other hospital.

The first paper is from the pen of that clever and indefatigable physician, Dr. Bright. The subject of investigation is obscure, and much of conjecture is necessarily mixed up in the series of cases given by our author—there being but few proofs exhibited by dissection. Dr. B. remarks, that patients very often dwell on one particular symptom, namely, a diffused pain over the occiput—often distinctly referred to the occipital nerve or a branch of the second cervical. The frequent occurrence of this symptom seems to Dr. B. to stamp it with sufficient importance to give it a claim to our attention in a diagnostic point of view. He thinks this symptom is often indicative of a diseased state of the cerebral arteries. A couple of cases will best illustrate this supposition.

*Case* 1. "A gentleman, past the meridian of life, after exposure, four years ago, first became the subject of an attack, which, by the description, appears to have been apoplexy from vascular congestion. Since that time he has been subject to various nervous affections;—vertigo; pain in the head, accompanied by sickness; palpitation of the heart; muscular weakness, particularly of the lower extremities; confusion of mind; and, latterly, a slight attack of hemiplegia of the left side, from which he has completely recovered. Still, however, the symptoms recur; and, amongst the rest, the most frequent complaint he makes, is, of what he calls a rheumatic pain at the back of the head, which he refers precisely to the space behind the mastoid process, spreading thence over the occiput. This pain sometimes occurs on both sides, but more frequently upon the right. He is, at times, exceedingly irritable, and his spirits very low; after which, he says, 'I feel completely knocked up, and have much pain in the back of my head, like a rheumatic pain, generally at the same spot, the right side of the back part of the head."

*Case* 2. A gentleman approaching the fiftieth year of his age, consulted me, a few years ago, for a pain, which he considered muscular, in the back of the head; an occasional feeling in the chest, as if the heart ceased to beat; and several nervous symptoms. I found that he had previously suffered one or two seizures, in which giddiness and momentary loss of recollection had occurred. I lost sight of him for a year or two; and, when he again consulted me, I found that, in the interval, he had experienced a serious attack of paralysis of the portio dura, on the left side; from which he was then very gradually recovering. But, amongst the other symptoms, that on which he laid the greatest stress, was his headache; which he described as being of two kinds; one occurring occasionally, which he considered common headache; the other much more frequently, and consisting of a pain in the posterior part of the scalp; and which, he said, he scarcely knew whether to consider as rheumatism or as headache. He pointed out the place occupied by this pain, most minutely; placing his finger just behind the mastoid process, and carrying it along the occiput."

There is probability, though not certainty, in the foregoing conclusions. In the following case, dissection came in to the support of theory.

In April, 1835, Dr. B. visited a lady, aged 56, labouring under symptoms of head affection, of nine months' duration. The headaches were attended by nausea, recurring occasionally till Dec. 1834, when she was salivated. Ten weeks previously to being seen, she had a suspicious attack of hemiplegia. On the 20th April, she came under treatment.

"She then complained of an obtuse pain at the top of the head, noise in the ears, and want of sleep: there was a wild expression in her countenance, and hesitation in her speech: the action of the heart was occasionally violent; pulse from 90 to 100, full and hard: tongue slightly furred: appetite not much impaired: continual nausea. The treatment adopted consisted of bleeding, which was twice repeated, to the extent of 12 or 15 ounces each time; leeches to the temples, perpetual blisters to the nape of the neck and behind the ears, evaporating lotions and free purging. The blood first drawn was not at all buffed; but the heart became irritable after its loss, beating with great force and frequency as she recovered from an approach to syncope: the blood drawn at the second bleeding was much cupped and buffed. The pain was not at all mitigated by the remedies, nor was the pulse much reduced in strength; and she only obtained slight relief by the use of extractum lactucæ, which was taken at bed-time, and occasionally in the morning."

On the 14th May she was reported as better, but still complained of pain near the occiput, corresponding with the egress of a large branch of the second cervical nerve. We need not trace the progress of the disease, or the list of remedies employed. She had two epileptic fits before she died.

There were several patches of fibrinous lymph on the external surface of the dura mater, the result of former inflammation. The whole of the vessels at the base of the brain were more or less diseased, being dilated, semi-opake, brittle, and studded with spots not quite ossified. Some other of the cerebral arteries were also diseased. The pons varolii was harder than usual. There was a small clot of blood, the size of a pea, in the right lobe of the brain. The pericardium was loaded with fat, as also the heart, which was hypertrophied in the left ventricle.

Our author now ventures one step farther in the thorny path of diagnosis, and assumes, "that in those cases where disease of the vessels is attended with unusual symptoms of lethargy, and the superficial pain of occiput is less observable, it is, in his opinion, probable that the disease has been situated chiefly in the internal carotids and their branches."

Dr. B. then relates a case where lethargic symptoms ran to a great degree, and for a long time, and where the carotids were found unusually diseased—the vertebrals being little altered.

The sixth case in this report was a gentleman aged 63, who had suffered for many years under cerebral affections, marked by pain and uneasiness diffused over the head—occasional coma, &c. relieved generally by bleeding and purging. After a kind of apoplectic seizure some hemiplegia was left, with confusion of thought. Another apoplectic attack carried him off, though slowly.

There was found a large quantity of serum under the arachnoid, which was thickened. The sulci were full. The brain was softened in several places. The lateral ventricles were distended with water. The vessels at the base of the brain were extensively diseased, by means of thick lumps of atheromatous deposits. The carotids and vertebrals were both diseased. Since his death, Dr. B. learnt that this gentleman was affected by lethargic somnolency for 12 years before he died. After detailing two or three other cases, Dr. B. sums up thus.

"The cases which I have thus brought together may be considered as presenting several of the different forms which disease of the brain assumes, in consequence of a morbid change gradually taking place in the arteries. Probably, while the vessels still remain entire, although diseased, they may give rise to general pain in the head internally;—and to pain, local or diffused, external to the skull;—or lethargic somnolency may be the prevailing symptom :—or apoplectic seizures may occur from simple congestion ;—or, by some process, possibly the interruption of the circulation in some part of the brain, a softening of its structure may be produced ;—or serum may be thrown out, either into the ventricles or under the arachnoid. When the vessels yield, it often happens, that single branches giving way, the shock upon the nervous system is almost momentary, or of short duration ; but probably some slight paralysis remains : such is the origin of the small cysts and cavities we occasionally discover. Should a larger vessel yield, or, by its diseased condition, cause a greater burden to be placed upon its branches than they can bear, the overwhelming apoplexy is the result. Now, of all these states, the foregoing cases are calculated to afford examples and illustrations."

In the present state of our knowledge we can only attach probability and not certainty to the inferences which Dr. Bright has drawn from the cases which he has detailed, leaving the question open to further enquiry and investigation.

### Permanent Contraction of all the Extremities. Numerous Cartilaginous Deposits on the Arachnoid of the Spine.

This is a very curious and distressing case related by Dr. Bright in the first number of Guy's Hospital Reports. A female, aged 28, was admitted into Guy's Hospital in Sept. 1835, incapable of standing or assisting herself. Her hands were contracted and bent downwards—her feet extended, and her toes bent backwards. The shoulder joints were flexible—the hands morbidly sensible—no tenderness, on pressure, along the spine—urine and fæces under control—no pain in the head—mind clear—bowels regular. Shortness of breath, and symptoms of chronic pulmonary affection. She stated that, three months previously she had slept in a damp bed, and to this she ascribed her malady. Rapid disease of lungs came on, of which she died in about two months.

The brain and spinal cord only were examined. A considerable effusion was found under the arachnoid. The brain was of healthy consistence, and free from disease. The theca of the spinal cord was thought to be a little opake—the whole arachnoid was transparent. "The only very obvious diseased appearances was the deposit, externally to the arachnoid, of a great number of flat cartilaginous bodies, looking like small portions of tallow dropped into water. Fifty or sixty of these were counted. They were situated chiefly at the lower part of the cord, and the commencement of the cauda equina. The vessels at the lower part of the spine were decidedly congested."

This is an obscure case, and it is very difficult to say whether the morbid appearances found on dissection were really the cause of the contractions of the limbs.

### An Essay on the Symptoms and Diagnosis of Pericarditis. By Dr. Hughes.

This Essay was read before the Physical Society of Guy's Hospital, and is now published in the first number of the Hospital Reports emanating from that celebrated school. Dr. H. informs us that this Essay is the result of "constant attendance upon the sick, and a close examination of the disease itself." These are the very best recommendations we can have.

He thinks it remarkable that a disease now so common as rheumatic pericarditis is, should have been unknown to the profession 30 years ago. It is so—but probably this, as well as some other diseases, have increased in frequency since the beginning of the present century. All diseases of the heart have, unquestionably, augmented in number and intensity since the French revolution. Even to this day, our continental neighbours seem to have very little idea that pericarditis owes its origin generally to rheumatism.

The diagnosis of pericarditis has hitherto not been settled. It has been found, on dissection, where it was not suspected in the living body—and it has been confidently predicted before death, when no trace of it was discovered *post-mortem.* Our best pathologists are not quite in accordance as to its diagnostic marks.

"At one time, we hear of, and witness, excessive præcordial anxiety; at another, it is scarcely observable by either the patient or physician; sometimes, we find severe local pain; but sometimes, as must be known to all who are moderately acquainted with it, the disease exists, even in its acutest form, without any. This pain is by some found to be increased on pressure, in almost all instances; while, by others, tenderness on pressure is said to be very rare. Here we observe extreme dyspnœa, orthopnœa, and thoracic distress—there they are almost entirely wanting; in one case we find a strong, sharp, thrilling pulse; and in another, a small, weak, irregular, and unequal one; which, by this author, is said to appear only during the progress of the complaint; and by that, to be present, from the very commencement. At one period, we are told that the hardest pulse known is present in pericarditis; and in a few years we are informed, by the very same man, that a small, weak, and intermitting one is a principal characteristic of the disease. This writer acquaints us, that here the stethoscope fails altogether, or affords but little aid; while by that we are informed that percussion and auscultation are the best, and sometimes the only means, of attaining a correct diagnosis. Sometimes the sufferer cannot lie on the right, sometimes not on the left, side; in this case, only on the back;, and in that, not at all. Some recent writers, indeed, assert, that the difficulty of ascertaining the presence of this important affection is entirely removed by modern discoveries and observations; and one of the latest affirms, that pericarditis may be distinguished with almost as much facility as pleurisy. I am still persuaded, that cases occur in which no characteristic symptoms are observable, or at least likely to be observed; that is, that pericarditis occasionally exists under circumstances, in which there appears nothing to direct the attention to the heart, and in which, therefore, though some physical signs might, if sought after, be positively discovered, there are no indications of the inflammation of its investing membrane. I think, therefore, I am still justified in saying, that the disease is sometimes present without symptoms; that some cases are only to be discovered by negative signs (*par voie d'exclusion*); and that there are others, and those a great majority, which are to be recognized by indications of a distinct and positive, though variable, character."

Of those cases in which there are no signs, positive or negative, Dr. Hughes acknowledges that he has seen none. Yet such cases are recorded by Latham, Stanley, Andral, Rostan, &c. In these cases, it would appear that the pericarditis was masked by affections of the brain and other organs—and probably the attention of the practitioners was drawn off from the real to the apparent seat of the disease.

"The next set of cases are much more common. They are generally marked, simply by a feeling of distress and oppression at the præcordia, increased on motion and speaking; incapability of lying down, dyspnœa, dry cough, restlessness, a countenance expressive of suffering and anxiety, and a frequent, small, and occasionally intermittent pulse. Such symptoms, it is evident, may, and frequently do, appear in serious affections of the lungs and pleura; in extensive latent pneumonia, for example, or copious and double pleuritic effusion. These cases are to be distinguished only by a diligent examination of the chest, by percussion and auscultation. It is only by first proving the absence of dulness on percussion, increased resonance of the voice, and crepitant rattle indicative of pneumonia, or of sharp pungent local pain, dulness, and the peculiar shrillness of the voice, called ægophony, characteristic of pleurisy, that we shall be enabled to pronounce the disease pericarditis. We may then be able, by a more minute investigation of the heart, to confirm our opinion; and, in the progress of the complaint, as fresh symptoms arise, or old ones become modified, to remove any remaining doubt. These cases are usually insidious in their approach, doubtful in their origin, slow in their progress, and uncertain in their termination.

The next set of cases, or those in which pericarditis may be discovered by positive and well-marked characters, are fortunately, by far, the most numerous. They are, however, as it has long appeared to me, clearly divisible into two groups; which usually arise from different causes; are marked by different, and, in many instances, even opposite symptoms; are followed by different results; and present different morbid appearances after death. This distinction, I believe, arises from the varying nature of the inflammatory effusion: for not only is it true, that, as observed by writers on this subject, solid plastic matter may be first effused, and, in the progress of the complaint, sero-purulent fluid may occupy its place; but it is likewise true, that the effusion may be, in a great degree at least, solid during the whole course of the disease; and that it may be purulent, or sero-purulent, from the very commencement. It is, moreover, from these facts having been lost sight of; from this distinction not having been noticed, or sufficiently borne in mind; from the effusion, at one time solid, and at another fluid, not having been regarded as producing different and often contrary symptoms, that has, I think, arisen — and that, up to the present time, has continued—a great portion of the uncertainty, obscurity, and confusion, connected with this complaint. Now, though some authors, as Dr. Hope, and Mr. Mayne, refer to the diversity of symptoms occasionally arising in the course of the disease from the change of the inflammatory deposition, I was not able to find any notice taken by authors of the distinction alluded to, as marking different complaints, till, a few months ago, I perused the admirable papers of Dr. Latham, in the Medical Gazette, and the essay of Dr. Stokes, a few years since, in the Dublin Medical Journal: each author makes a distinct, though brief reference to the varying aspect of different cases as arising from this cause. While, then, I must confess that I was rather disappointed at finding myself, in some degree, forestalled on a point that had engaged my attention for some years, I felt gratified at seeing my own observations confirmed by the practical knowledge and extended experience of the gentlemen just mentioned.—In those cases which are attended with sero-purulent effusion, the symptoms are severe, from the very commencement; they rapidly increase, and, unless checked by prompt and active treatment, speedily terminate in death. The inflammatory fever, from which the patient suffers, is active only, for a short time, and quickly assumes an asthenic character, or altogether disappears. He sometimes complains of acute pain, occasionally shooting to the shoulders and back, but more frequently of a severe burning heat in the region of the heart, with a sense of weight and intolerable oppression at the præcordia: he is unable to lie upon either side, and sometimes even upon the back, but is obliged to sit erect, or inclined forwards, with the hand pressed to the affected side; though restlessness, and vigilance are constant, yet, whatever be the position, it is constrained, as slight motion threatens suffocation, and any attempt, or disposition, to sleep is quickly followed by imminent danger of choking. The respiration is hurried, gasping, and thoracic; there is a frequent feeling of faintness, and sometimes syncope itself; palpitation, though almost continual, occurs in increasingly violent paroxysms; the countenance is expressive of distress, anxiety, and fright; the alæ nasi are distended, the eyelids widely separated; the face is bedewed with perspiration: soon the lips and cheeks acquire a purple tint, and the extremities become cold and clammy. The jugulars are distended, and undulate; the carotids doing scarcely more, as the pulse is very small, frequent, intermittent, irregular, and unequal. The impulse of the heart is generally much decreased, and often scarcely perceptible; the hand placed over it being sensible merely of a diffused, variable, and undulatory motion, with an occasional strong beat: the sounds are distinct and low, but usually soft and pure. The rhythm is in the highest degree confused. On percussion, the natural dulness of the region is found considerably augmented, both in extent and degree, perpendicularly, as well as transversely; and, after a short period, a fulness or projection is said to be observable; a symptom which, I must confess, I have not hitherto witnessed in acute pericarditis; though, in the chronic form of the complaint, as well as in hydropericardium, it is, I believe, not uncommon."

When the above symptoms have *suddenly supervened,* we may confidently predict that pericarditis, with considerable effusion, has taken place. These things generally occur in weak constitutions. The disease may be confounded with inflammation of the præcordial and diaphragmatic portion of the pleura; but by careful attention to the symptoms a discrimination may be made.

In the other group of cases, the disease is more slow—the symptoms less alarming—and the termination more favourable. The patient may live for weeks, and then recover, even when little or nothing has been done in the way of remedies. The fever is sthenic, the skin hot, and the face flushed. The præcordial pain is more acute, and is usually increased on pressure or coughing. The patient cannot lie on the left side, but reposes easily on his back. The respiration is frequent, and chiefly thoracic, but without heaving or labour. He moves and talks without inconvenience, and his features are unaltered. The pulse is frequent, tense, and jerking, but regular. The normal præcordial dulness is not increased. The impulse of the heart is sharp, but not strong and heaving, as in hypertrophy.

"Now, it must be obvious to all, that the symptoms last related are extremely different from, and in many particulars opposed to, those previously mentioned; yet are they the true and genuine symptoms of pericarditis: but they are symptoms of pericarditis with solid or plastic effusion; symptoms, moreover, which are sufficient to distinguish it, not only from that with serous, sero-purulent, or purulent effusion, but also from every other complaint, and thus to render clear the diagnosis of this form of the disease. It is true that the dry or plastic form of pleurisy presents the same frottement or rubbing, &c.; but an obvious mark of distinction exists in the synchronism of the one sound with the action of the heart, and of the other with the respiratory movements."

"Now, according to my own observation, the pericarditis, that so constantly accompanies, or follows, rheumatism affecting young subjects, belongs uniformly to the latter, or dry form of the complaint; though I am aware that a few cases, related by authors, sufficiently prove that it is not universally so. Still, taking a view of the cases upon record, or any separate portion of them, and examining them with reference to this particular point, no one can, I think, fail to observe, that the number in which rheumatic pericarditis has been accompanied by fluid, and particularly by sero-purulent effusion, is extremely small."

Dr. Latham, in his lectures, has alluded to this solid effusion, in the following words.

"In the pericarditis that accompanies rheumatism, the symptoms during life are those that have been mentioned as strongly indicating the predominance of the solid over the fluid products of inflammation; and here dissection after death uniformly discovers solid lymph upon the pericardium, with very general or complete adhesion."*

Our author thinks that the "*bruit de soufflet,*" so early in its appearance and constant in its duration, arises from inflammatory thickening of, or deposition on the fibrous tissue surrounding and entering into the composition of the valves of the heart—coincident with, or perhaps preceding the affection of the external membranes. In this he differs from Hope and Stokes; but we think it unnecessary to discuss this point. The following are the conclu-

* Lectures in the Medical Gazette.

sions of our author—and the conclusion of the paper.

"1. Pericarditis occasionally, though rarely, occurs without any obvious, sometimes with only negative, but most frequently with positive and well-marked symptoms.

2. The cases, with characteristic signs, of which the diagnosis is not difficult, are of two kinds which are attended by different and even opposite symptoms, and generally followed by different results.

3. This difference of character depends upon the nature of the inflammatory product; which is fluid in the one, and in the other, for the most part, solid.

4. The cases with fluid effusion are generally rapid in their course, and fatal in their result; while those with solid effusion often continue for a long period, and the patients frequently recover, when little or nothing is done for their relief.

5. Though generally continuing of the same character during the whole course of the acute complaint, yet, as the nature of the effusion may change in the progress of the disease, these different sets of symptoms may both appear, at different times, in one and the same case.

6. Rheumatic pericarditis is almost universally accompanied with solid effusion, pain in the præcordia increased on pressure, regular and sharp pulse, and *bruit de soufflet*.

7. This *bruit* is, in these cases, probably dependent on inflammatory thickening of the valves, or of the fibrous tissue connected with them."

On the first blush, a formal Essay on pericarditis, which had been read and discussed in a medical society, seems rather oddly placed in a volume of hospital reports; but the surprise vanishes when we find that the facts on which the Essay rests have been collected within the walls of the institution, and may, therefore, be properly considered as part and parcel of the practice of it. There can be no doubt that this Essay proves the author to be a gentleman of accurate observation, and though he will seem to have refined a little, his conclusions deserve the respectful attention of the profession.

---

### St. Thomas's Hospital.

### Clinical Reports. By J. South, Esq. Assistant-Surgeon.

We noticed these reports in our last Number, but we had not time nor space to complete our account of them. We may now state that the pamphlet, for it is one, consists of 118 pages, and that it contains eleven reports, on the following subjects: — An Introductory Clinical Lecture, delivered by Mr. Travers—Syphilitic Eruptions, treated with Hydriodate of Potass—some Cases of Wounded Arteries—Extraction of a broken Catheter from the Bladder, by the Urethra—Mortification of the Hand and Forearm, following Contusion—Dropsy, terminating in profuse diuresis—Retention of Urine, arising from Gonorrhœa—simple Fracture of the Leg, followed by Loss of Motion of the Right Side of the Head and Face, and of the Arm, from Deprivation of Stimulus—Hydrocele treated by the Seton—Observations on Delirium Tremens, by Dr. Roots—Absorption of a portion of the Cranium and Cerebral Disorder, from a Contused Wound.

Such are the contents of the pamphlet before us. Five out of the eleven reports were analysed in our last Number. We have not space for the whole of the remainder at present, but shall select the following:—on wounded arteries—extraction of a broken catheter from the bladder—retention of urine from gonorrhœa:—fracture of the leg succeeded by loss of motion of one side. The cases of dropsy and of mortification of the hand and forearm must be deferred for our succeeding Number.

### I. Two Cases of Wounded Arteries.

Case 1.—*Wound of the Brachial Artery in Venæsection.*

A married woman, aged 25, was bled on the 3d of March, 1834, whilst pregnant, and in the operation the brachial artery was wounded. She lost eight or ten ounces of blood, when she became faint and extremely hysterical. Soon after her admision, a ring tourniquet* was put on so as to compress the artery near the insertion of the coraco-brachialis; a firm compress, wetted with cold water, was applied over the wound; the limb bandaged tightly from the fingers to the tourniquet, and the arm elevated, so as to favour the return of the blood to the heart. The whole limb was enveloped in flannel. She was ordered to take *Ammon. carb. ex Mist. camph.*, if the hysterical affection should continue.

A dose of the ammonia had the desired effect, and nothing of consequence occurred until the 7th, when, the ring tourniquet being found to slip, a common tourniquet was substituted for it. In changing the compress and bandage, a tea-spoonful of healthy pus made its escape from the puncture. She had previously complained of pain in the arm, which this discharge relieved. Some simple dressing was applied; over it a soft compress of lint; and the whole secured by a slight bandage. A common arm-splint was placed on the under surface of the limb, to keep the arm extended.

There was some pyrexia, and the bowels were confined. These symptoms were relieved by appropriate remedies. On the 8th, the tourniquet was slackened considerably, yet no hæmorrhage ensued. On the 10th, the tourniquet, producing great pain, was removed altogether. The wound was granulating. We need not say more, than that the wound healed perfectly: on the 10th of April, the artery could be felt pulsating in its natural situation, and to all appearance in its natural state. On the 16th the patient was dismissed cured.

The following short clinical remarks by Mr. Tyrrell are highly deserving of attention.

"I have seen the patient frequently since; and she can use her arm without difficulty, even in heavy work. There is no appearance of disease of the artery or veins. This is the fifth case of puncture of the brachial artery in bleeding which I have treated on the plan and principle here described, and in every instance has there been a successful result; no uneasiness, no varicose aneurism, no aneurismal varix forming, but the limbs apparently as perfect as if such accident had not happened. In one case I had an opportunity of ascertaining the effect of the treatment several weeks after the cure had been completed. A patient, under the care of one of our physicians at St. Thomas's, was the subject of some organic cardiac disease, for the relief of which venesection was ordered, and in performing this operation, one of my dressers punctured the brachial artery; I was sent for, and adopted the treatment prescribed in the foregoing case, and with success. Some weeks after, the patient died suddenly in the hospital, when I carefully examined the arm, and found that the median vein had its canal obliterated for half an inch above and below the puncture; it was adherent to the artery, the wound of which was firmly united, and its calibre perfect. The parts were removed, and are preserved in the museum of our hospital."

Case 2.—*Wound of the Radial Artery.* A man wounded the radial artery at

* The ring tourniquet consists of a metal ring, having a diameter larger than that of the limb to which it is to be applied, and a width of about an inch; the circumference is tapped at one point, so as to admit a screw, to the inner extremity of which a pad is fixed, and to the outer end a small handle to turn the screw with, by the action of which the pad can be carried to or from the centre of the circle. When applied, this instrument makes pressure only on two parts; by the pad on the site of the artery, and by the portion of the ring immediately opposed to the pad, on the surface of the limb directly opposed to the position of the artery: thus it does not interfere with the lateral circulation.

the wrist with a penknife. He arrested the hæmorrhage by means of pressure until he arrived at the hospital. There pressure was made by cork upon the wound, and compresses and bandages were applied to it. The pressure occasioned pain and œdema of the hand, and when the compression was lessened, six days afterwards, bleeding again occurred. Fresh pressure was consequently necessary. The patient was not dismissed, for the wound was not quite healed, till nearly a month after the accident.

This was evidently bad practice, and Mr. Tyrrell admits that it was so.* The artery, situated so superficially, should have been secured at once above and below the puncture. But Mr. Tyrrell points out what he deems the best method of treating the wounds of arteries deeply seated, and difficult to be got at by an operation. The principle, a very rational one, consists in arresting or diminishing the flow of blood to the injured vessel, by pressing on the trunk or trunks communicating with it; in keeping the wounded parts carefully adapted by moderate pressure ; in preventing venous congestion, by position and support of bandage, and in forming the adhesive union by perfect quiet. To effect a cure, it is necessary to persevere in the treatment for two or three weeks.

We think these brief suggestions well worthy of attention. We have no doubt that, in many instances, compression of wounded arteries proves useless or injurious, only because it is improperly applied. There is no reason, abstractedly speaking, why the fine punctured wound of the brachial artery inflicted by a lancet, should not heal. In Mr. Tyrrell's cases, and in several which have been published by Mr. Colles and others, the wound, under appropriate pressure, *has* healed. The too frequent failures are owing, we are confident, to the trepidation or unskilfulness of the surgeon.

* The patient was, in the first instance, under the care of the dresser.

### II. Extraction of a Fragment of Catheter from the Bladder, through the Urethra.

The following case is creditable in the highest degree to Mr. Tyrrell. It well deserves to be recorded.

*Case.* A man, aged 40, who had for some years suffered from stricture, introduced into his bladder a catheter, which was so worn and weak at the curve as to have been soldered there. The instrument met with some obstruction at the bulb, and broke at the weakened part. The fragment could not be withdrawn, and the man set out from Reigate to St. Thomas's Hospital, walking a considerable part of the distance. When admitted, he did not suffer very much. Mr. Tyrrell was sent for, and the following is his own account of what he found and did.

" On passing a sound, I discovered the piece of catheter lodged at the fundus of the bladder, transversely; its extremities being embraced by that viscus, so as to be held with some firmness. I dislodged it by passing the end of the sound beyond it, and, drawing it forwards, moved it to the lower part of the bladder behind the prostate gland; where by sounding I ascertained that its position was still transverse. From the freedom and extent to which I could move the sound, I concluded that the bladder contained several ounces of urine, and that it was sufficiently distended to afford good opportunity for conducting the plan I had decided to adopt for extracting the piece of catheter.

The patient was placed on a bed in a half-sitting posture, with his thighs semi-flexed; the sound was withdrawn, and I then introduced one of Weiss's instruments for extracting small calculi, which was nearly straight, and had a strong spring. By careful examination with it, I discovered that the extremity of the foreign body towards the patient's right side was free, and that the other was covered with a fold of the bladder. After several unsuccessful attempts, I succeeded in seizing the free extremity with the instrument, and, by withdrawing it very cautiously, I brought the piece of catheter into the urethra, when the forceps slipped from it. I immediately introduced my finger into the rectum, for the purpose of compressing the urethra between the foreign body and the bladder, so as to prevent any retrogade movement of the former. This being secured, I again introduced the forceps into the urethra; and in the first attempt caught the piece of catheter, and drew it out.

The portion of catheter removed, measured exactly three inches in length, and was of the size of No. 10 of Weiss's gage.

The examination and operation together occupied about twenty minutes."

A dose of opium, and one of castor oil were all that were required. Three days after his application at the hospital he was dismissed well.

This case is deserving in a high degree of the attention of our surgical readers. As Mr. Tyrrell observes, there is probably no other instance on record where a portion of catheter has been thus extracted from the bladder. At all events surgeons, remembering this case, should hesitate before they have recourse to the lateral operation of lithotomy, for the removal of bodies of this description.

### III. Retention of Urine arising from Gonorrhœa.

*Case.* A man, aged 35, accustomed to drink spirits freely, contracted gonorrhœa. For this he drank eight glasses of gin-and-water daily. On the 7th day he was unable to make water; so he augmented his potations. On the 8th day he could still pass no water, but had a constant desire to do so. The bladder was greatly distended. He was now received into the hospital.

*V. S. ad* ℥ xij. *(ad syncopen.) Ol. Ricini. Suppositor. Opii, gr.* ii. *Hirud.* xlviii. *perineo et peni. Fotus.*

In the afternoon his bowels were opened, and he passed half a pint of urine. In the evening, however, the bladder was much distended, and the patient was in great pain. An attempt was made to introduce an elastic catheter smeared with belladonna, but the agony it occasioned was so great that the instrument could not be passed. After fomentations the patient voided about a pint of high-coloured urine. *Warm bath—diaphoretics.*

Next day he passed two half pints of urine and was easier; but the bladder was still distended. *Hirud.* xx. *regioni pubis.* In the evening he was still much distressed by the retention, having voided a pint of water only. *Hirud.* xxiv. *corp. spongios. Hip bath. Extract. belladonnæ applied.*

On the following day he was no better, and a small catheter was introduced with very great pain; three pints of urine were drawn off.

*Liq. Op. Sed.* ♏ xij. *Liq. Potassæ,* ♏ xv. *4tis horis.*

After this he gradually regained the power of emptying his bladder. In a week he was discharged. He had then only a little pain in micturition.

We observe no mention of any examination of the bladder, through the rectum, having been made in this case. Yet it is highly important, and the surgeon should never neglect to ascertain precisely the condition of the neck of the organ, and of the prostate. Whilst the patient was suffering extreme pain and unable to empty his bladder, he was ordered to take the balsam of copaiba. We cannot say that we like this practice. Suppose there had been abscess forming in the prostate, a supposition by no means unlikely, the copaiba must have occasioned mischief. It is this indiscriminate employment of stimulating remedies in gonorrhœa that we deprecate. Some little time ago, we saw a gentleman who was ordered to take cubebs, while in the state of the patient whose case has just been related. The first dose or two seemed to do him good, when suddenly the most furious inflammation of the mucous membrane of the bladder succeeded, and a very protracted illness was the consequence.*

### IV. Simple Fracture of the Leg, followed by loss of motion of the side of the head, &c.

*Case.* A stout young man of intempe-

* The reader may be referred to rate habits, slipped, when intoxicated, and broke both bones of the left leg, at a part where they had already been five times fractured. A good deal of spasm occurred at first. Eleven days after the fracture he complained of headache, and want of sleep. Purgatives were exhibited. On the 13th day, the headache continued, he was heavy, his head was hot, and his hand was so unsteady that he dropped the basin, while eating his dinner. On the 14th day, there was partial loss of motion in the left arm, entire loss of motion on the left side of the face, and traction of the tongue *towards* that side. The pulse was 72, and bore pressure.

*C. C. Capiti ad* ℥ xvj. *Abras. capill. dein. Lot. frig. Rep. Pulv. jalap.* c̄. *Hyd. sub.*

On the 15th day, answered questions better—pain in the head diminished—tongue protruded in a straight line—pulse less powerful.

*Hirud.* xx. *tempori.*

On the 17th day, sleeplessness—frequent delirium—no heat of the head or surface—pulse irregular and very compressible—bowels open.

*Ordered a pint of porter and four ounces of gin daily.*

On the next day he was much better. On the 19th day, the delirium was gone. On the 22d day, the paralytic symptoms had disappeared. At the end of the sixth week the fracture was found to be perfectly united.

This case, very interesting and very important as it evidently is to the practical surgeon, afforded Mr. Tyrrell, under whose care it was, an opportunity of making some clinical remarks to which we would particularly direct attention. The clinical observations in question refer to two separate points—the frequency of fractures in the same individual, and the removal of delirium and paralysis by stimulants.

1. This patient broke the same leg six times, and the other leg once. Yet his occupation did not appear to be one, which would render him particularly liable to fractures of the lower limbs. We must suppose that in him there was more than usual brittleness of bone. As an illustration, Mr. Tyrrell cites a case which came under his own observation.

"It is unusual to find, at the middle period of life, many features occurring in the same individual. I have, however, had under my care a patient several times who is a remarkable instance of the brittleness of bone, or what is termed *fragilitas ossium.* He had been the subject, at the time he was last under my hands, of seventeen fractures; and when I last saw him three or four years ago, he had had five more fractures, making in all twenty-two. These fractures affected the femur, the tibia and fibula, the upper-arm and the fore-arm — scarcely a cylindrical bone of any size had escaped. In consequence of these fractures he has lost in height from seven to eight inches. The first time I had him under my care was in consequence of fracture of the thigh bone, and the other had been fractured once or twice previously. In consequence of indifferent surgery, that limb was shorter by three inches than that for which he came under my care. He had worn an iron to make up the difference in length of the two limbs, and it enabled him to make progression with some inconvenience. Finding this, I stated that it was possible to set the recently-broken limb to the same length as the one formerly broken, and at his wish I did so. I made an angular union of the second limb, reduced it to the same length as the other, and he was enabled afterwards to make progression more easily and rapidly. Hence I was the instrument of taking off three or four inches from his height, by shortening the limb to that extent."

Mr. Tyrrell makes the observation which has been made by other surgeons, that in the cases in which this brittleness of the bones exists, there is an almost equal facility of union. This is a curious circumstance.

2. Mr. Tyrrell draws attention to the occurrence of loss of motion of the tongue, side of the face, and left

---

some observations on this subject by Mr. Henry James Johnson, at page 488, of the present number.

upper extremity, as a consequence of the deprivation of stimulus. He observes that this need not excite astonishment, when we reflect, that the brain may cease to execute or imperfectly perform its functions, from want of blood, as well as from excess of it. We see this to a marked degree in syncope. Mr. Tyrrell saw a case in which, in consequence of "great exhaustion of the vital fluid," blindness of one eye occurred, but disappeared after the administration of stimulating or tonic medicine.

Mr. Tyrrell alludes to several cases, in which delirium, and other symptoms, were relieved by the exhibition of the stimulants to which the patient had been previously habituated. The attention which the characters and treatment of delirium tremens have of late received, render a particular notice of many of Mr. Tyrrell's observations, and of some of the facts that he relates, unnecessary. But the following is worth recording.

"I admitted a man with symptoms of concussion, so much so that when he came to the Hospital reaction had not taken place; he was still in a collapse, with loss of sense and voluntary motion: the pupils were dilated. In this state warmth was applied; he was placed in a recumbent posture, and by degrees reaction took place. He suffered severely afterwards from symptoms of *meningitis*, (inflammation of the membranes of the brain,) so that he was obliged to be depleted and mercurialised, to a considerable extent, to check it.

He had a wound of the scalp at the time of his admission, and during the first part of the treatment this wound went on well. It was a contused wound and not likely to unite by adhesion; but it began to granulate and suppurate healthily. He became the subject of erysipelas, and he became delirious, raving more violently than either of the patients whose cases I have alluded to. I was called to see him in this state. Upon examining the condition of the circulation, I found it was irregular, as in the two preceding cases. It struck me that he might be suffering under a deprivation of stimuli. I found he had been accustomed to take freely both of spirits and beer; I still, however, (it being the first case I had seen where such an affection had followed injury of the head,) did not like to give strong stimuli. I thought I would be cautious in my proceeding, for the symptoms might arise from a repetition of mischief of an inflammatory kind in the brain, as well as from a deprivation of stimulus or irregularity of the circulation. I therefore gave him a dose of carbonate of ammonia (10 grains,) and desired that if he became more tranquil under the influence of this, that, in the afternoon, the dresser should give porter or a small quantity of spirits. Instead, however, of following up that plan of treatment which I had pointed out, and on which I thought I had sufficiently explained myself to those who accompanied me on the visit, the dresser sought the assistance of one of my colleagues who was in the Hospital, but he did not tell him my opinion of the case—he only said that he wished him to examine a patient suffering from erysipelas of the scalp. The secretion of fluid was arrested, the part was puffy, as if there was fluid or air in the cellular tissue.

My colleague, thinking that there was matter at some depth, made a free incision through the distended or puffy part. In doing this he divided a branch of the occipital artery, and the patient lost about fourteen ounces of blood. Instead of being relieved, he became more violent—passed a restless night, and when I went to visit him in the morning I found an increased state of delirium and excitement. The pulse was more feeble than on the preceding day; more irregular and more of a depressed character than in either of the other patients. I immediately gave him some stimulus, but expressed it as my opinion that the case was beyond the reach of science or art, for I thought he was in that state in which effusion was taking place. He was comatose in a degree when left to himself, and when roused was extremely irritable.

He went to sleep soon after taking the stimulus; he slept tranquilly, and awoke without the slightest pain or inconvenience, but still disposed to sleep. His countenance was pallid and his circulation extremely feeble. Seeing him in this condition, I stated that the opinion I had previously formed was more confirmed; that the case was lost; that at the time I ordered stimulants, effusion had commenced, was going on, and would terminate his life. It was my opinion that stimulants could not be given in a greater proportion than I had administered them, without increasing the circulation to an injurious extent.

The case terminated fatally that day; the patient gradually became more and more depressed, and eventually died. I stated, before the *post mortem* examination, that I believed that effusion would be found of a serous kind into the ventricles at the base of the brain; that the brain would be pallid and the vessels be nearly empty from the loss of red blood. This proved to be the fact: we found serous effusion in the ventricles, and the brain unusually pallid, as if deprived of a large quantity of red fluid."

Of course there is no reason why exhaustion should not occur in irritable and intemperate habits, after inflammation of the brain, as after other affections. But it is exceedingly difficult to determine the real nature of the symptoms in these cases. We have several times seen what appeared to be delirium tremens after scalp-wounds and erysipelas of the head, and we must say that great caution and extreme judgment are necessary in forming any decisive opinion on the case or on its treatment.

We once saw a case of convulsions occur under circumstances of this description. An intemperate man received a scalp-wound. Erysipelas of the head succeeded, and for this he was purged and kept low. Suddenly he was attacked with violent convulsions. It was supposed that matter might be forming between the bone and dura mater. He was accordingly trepanned opposite the scalp-wound. The dura mater was perfectly healthy. Mild nourishment, and some trifling remedies were given, in the absence of any positive data with respect to the nature of the case; and the patient did perfectly well.

The following is a good instance of the necessity for exhibiting adequate support after even what seem to be trivial injuries.

" I operated on a man between 60 and 70 years of age, and extracted a cataract, but his powers were more feeble than usual at that age. I operated on the Friday, and on the Sunday the apothecary came to say that the man was complaining of pain. I found that the apothecary had employed leeches and blisters, the ordinary means for subduing inflammatory action, but seeing that the patient's countenance was pallid, his hand cold and the pulse irregular, I decided upon the propriety of giving stimulants, in spite of local pain, redness, and swelling. I ordered him a dinner of good broth, meat and beer, and also a small quantity of spirits. On Tuesday, when I paid the usual visit with the pupils, I stated the nature of the case—the remedies which had been employed, and what I expected would be their influence, viz., that it was very probable that they would save the patient's eye. We went up to see the man, and, rather to my surprise, the eye was free from redness, the section was united, and the eye appeared as if no untoward symptom had followed the operation. The eye was saved entirely by the appropriate use of stimuli; had I resorted to depletion, suppuration would have taken place; the organ would have been lost."

We do not quite agree with Mr. Tyrrell upon all points. He appears to deprecate depletion of every description almost, if not absolutely indiscriminately. We have seen a good deal of delirium tremens, and we are satisfied that it is often very advisable to commence with local depletion, by cupping, and to employ counter-irritation, although at the very moment, or directly afterwards, we may give narcotics and stimulants. It is not in all cases easy to determine whether there is, or is not increased action in the head, and where the latter is hot, with a full pulse, and a stout habit of body, we are satisfied of the propriety of feeling our way with cupping, cold to the head, and even blisters. We may be pretty sure that these measures will not kill a patient, and they will greatly simplify the symptoms, and render the indications of treatment more clear. More than once we have seen patients stimulated for delirium tremens, and that by judicious and well-informed men, and after death we have seen unequivocal traces of inflammatory action about the brain or its membranes.

In the case which forms the subject of the present report, we are not sure that the cupping, &c. in the commencement, did not pave the way for the successful employment of the stimulants afterwards.

---

### Edinburgh Royal Infirmary.

### Clinical Report for the Winter Session, 1834-35. By Mr. Syme.

This Report, which is published in our northern contemporary,* contains many cases which present no peculiar features, either of interest or of importance. Cases, for example, are detailed, where ulcers, having certain characters of malignancy, were removed, and the patients left the hospital well. Such cases prove nothing, interest nobody, and are rather calculated to injure science than to benefit it, because they affect to offer instances of the cure by operation of malignant changes of structure, when sufficient time has not elapsed, to permit any accurate conclusions to be drawn. These cases we need not and shall not notice.

#### I. Excision of the Superior Maxillary Bone.

The superior maxillary bone has latterly been several times subjected to operations, more or less of it having been excised for the cure of malignant disease affecting it. The following cases bearing on the subject may be cited.

*Case.* A woman aged 42, was admitted on the 26th of November 1834, with the cheek considerably distended by a tumor springing from the superior maxilla, which, though firm, did not possess the hardness of bone. When the finger was drawn along the lower margin of the orbit, an inequality in its surface was detected, and the floor of the cavity could be felt distinctly elevated. The palate, throughout the whole of its extent on the left side, and also for some distance beyond the mesial plane, was greatly thickened, and extremely irregular on its surface, which exhibited the characters of a malignant ulcer. She stated that ten years previously, she received a kick on the face from a cow, which was followed by swelling that never entirely disappeared. In the beginning of last year she began to suffer pain in the seat of enlargement, and at the same time remarked a great increase in the rapidity of growth. The superior molar and bicuspid teeth of the side affected soon afterwards loosened and came away. Within the last few months the progress of the disease had not been so rapid. The patient's health was good, and on the 28th, Mr. Syme proceeded to remove the superior maxillary bone.

"The patient being seated on a chair, a perpendicular incision was made from the inner angle of the eye down through the lip, and another from the convexity of the malar bone to the angle of the mouth. The flap thus formed was dissected up, and the integuments on each side turned back so as to expose the whole surface of the maxillary bone. One blade of the cutting-pliers was then introduced into the nostril, and the other into the orbit, so as to divide the ascending nasal process. A notch was next made with a saw in the malar protuberance, which then readily yielded to the pliers. After this, only the palate and septum of the nose remained to be divided, which was done by first circumscribing the morbid surface in the roof of the mouth with a sharp-pointed straight bistoury, and then cutting through the bone with the pliers. The diseased mass was now easily turned out to the side, and detached from its

---

* July, 1835.

connections, when it appeared that the tumour had been removed quite entire. It was of moderately firm consistence, and of a yellowish colour, springing from the maxillary bone, filling the antrum, and, by its pressure, had caused absorption as well as displacement of the floor of the orbit. The arteries that required ligature having been tied, the patient was conveyed to bed. An hour after the operation, the cut edges of the integuments were brought into accurate contact by stitches of the interrupted suture, except at the two points where the lip was divided, each of which was secured by the twisted suture, a sewing needle being used for the purpose. Cloths moistened with cold water were diligently applied. The wounds healed by the first intention, and the patient was dismissed on the 20th of December with wonderfully little deformity. She continues perfectly well."

As evidence that occasionally this operation is successful, Mr. Syme refers to the case which he published in one of his reports for 1830. The bone was removed for a soft, red, malignant-looking tumor. The woman still continues perfectly well.

Mr. Syme particularly enjoins the surgeon not to meddle with those cases in which the disease involves the bones of the nose or the base of the skull. To determine this, Mr. S. would seem chiefly to depend upon the history. He also enjoins the surgeon not to be induced to operate in cases in which there is merely a cyst containing fluid developed in the substance of the bone. The diagnostic mark of this he sets down, as a yielding and crackling, like parchment, when the bone is pressed on with the fingers.

We must remark, however, that this certainly is not a diagnostic sign of the presence of a mere cyst. In a case which we witnessed, the antrum was occupied by a true malignant tumor, yet the partial absorption of the osseous tissue of its anterior wall, gave rise to the symptom in question. Mr. Syme relates the following interesting cases which have come under his observation.

"A gentleman came from the country to have a tumour of the upper jaw removed. It depressed the palate, encroached upon the nostril, and elevated the cheek. At the most projecting part in each of these situations the tumour, though elsewhere of bony consistence, yielded to pressure, and recovered from it with a crackling sensation. Dr. Thomson, Mr. Turner, and I, who saw the patient together, agreed that his complaint depended merely upon a cyst, which would contract and be obliterated if freely opened. I accordingly evacuated a quantity of limpid fluid by making a puncture under the lip, after which the swelling contracted, at first rapidly, and afterwards more slowly, until it ceased to occasion any annoyance. Last Summer a young lady from North Shields applied to me on account of a tumour of the lower jaw, which extended from the bicuspid teeth to the articulation. It was round and firm, and had been growing for three years, having been preceded by pain in the bone, which led to the extraction of the grinders at the part affected. In the most prominent point of the tumour, where it distended the cheek, and also in the situation of the gum, which was expanded and flattened, I detected the characteristic yielding and resiliency of the thin osseous, or rather at these parts membranous, shell of a cyst, and did not hesitate to open the tumour by removing an elliptical slice from the alveolar region sufficiently large to prevent any chance of the aperture soon closing. Half a tumblerful of fluid, containing numerous shining scales of cholesterine, was evacuated. The patient suffered no inconvenience, and before long observed a remarkable diminution in the size of the swelling, which I understand has since gradually become smaller, and now does not equal that of a pigeon's egg."

Mr. S. makes the following remarks upon the treatment of these serous cysts of the maxillary bones.

"In such cases it has been advised to employ setons, caustics, and other violent means to destroy the secreting action of the cyst, and induce a healing granulation of the surface; but there is reason to regard this practice as unnecessary and hurtful. That it is unnecessary will appear from the cases just related, to which others attended with a similar result might have been added; and that it is injurious seems probable from the following one. A young man had a large tumour of the lower jaw, which was ascertained to depend on a cyst in the bone. A seton was passed across it from the mouth through the cheek. Contraction followed; but a solid growth afterwards occupied the place of the former cavity. My opinion being then asked, I advised excision of the affected portion of the jaw, which was executed by the gentleman who had operated in the first instance."

We would observe, however, that as mere puncture of a cyst very seldom succeeds in effecting a cure when the cyst is situated in the soft parts, we do not see why it should be more successful when the cyst is placed in the bone. It would be strange if here, where the circumstances favouring contraction are diminished, a cure should more readily occur. Still Mr. Syme's experience should be recollected.

On the whole, the cases of excision of the superior maxillary bone that have come before the public, are sufficient to shew that, in some instances, it has been successful, and has probably saved the patient from otherwise inevitable destruction. This is consistent with what we might anticipate, when we reflect on the morbid growths which affect the lower jaw, and on the occasional success from the partial or total removal of the latter.

At the same time, modern experience has run so counter to the indiscriminate or, indeed, to the general performance of operations, for the removal of malignant tumors, that the surgeon should pause before he rashly undertakes so painful, disgusting, and dangerous an operation, as the removal of the upper jaw for malignant tumors springing from it. He should weigh well the circumstances of the individual case—the nature of the morbid growth—its stage—its precise seat—and the general health and strength of the patient. On his discrimination and determination lies no slight load of responsibility—responsibility both to his patient and his art. We say to his art, because bloody and barbarous operations, if useless, redound to its disgrace. We have endeavoured to discountenance those butcher-like displays, so gratifying to low and sanguinary dispositions—on which men with better hands than heads rely for carving out a mechanical reputation—and which, while they make the vulgar stare, must make the judicious grieve.

Formerly, a surgeon was estimated, like a tomahawk-man, by the scalps of victims that he could exhibit. If he was a first-rate operator, he was then a first-rate surgeon. The physicians prescribed and he manipulated. Surgery was in its infancy only. Now the mere operator is shunned as a dangerous, not consulted as an able man. The triumph of our science is in preventing the necessity for the employment of cold steel—in discriminating the varieties of local diseases—and in determining their connexion with general constitutional derangement. For one case in the practice of surgery, in which mechanical dexterity is requisite, there are a hundred, aye many hundreds, in which mental qualities are necessary.

### II. ENCYSTED TUMOR IN THE NECK, CURED BY THE SETON.

*Case.* A man, aged 40, was admitted on the 1st of March, on account of a large tumor on the right side of the neck. It was nearly twice the size of a turkey's egg, and lay under the sterno-mastoid, before and behind which it projected. The swelling was tense, and, when carefully examined by pressure, allowed fluctuation to be felt distinctly, but no pulsation. It had first appeared after a fall upon the head, eight months previously. It had once been punctured, when a large quantity of fluid escaped.

Mr. Syme introduced a trocar at the anterior and lower part of the swelling, and drew off a quantity of thin watery-looking fluid, with scales floating in it. A seton was then conveyed through the opening already made, under the sterno-mastoid, and out behind it, so as to cross the tumor at its longest diameter. No remarkable consequence, either local or general, followed, and the seton, which had been changed daily, was withdrawn on the 12th. The upper and posterior orifice closed soon afterwards, and the lower one seeming insufficient for discharging the matter, which was copious and fetid, was repeatedly dilated with the knife, and, lastly, by the caustic potass. The swelling under this treatment gradually contracted, and the patient was dismissed on the 16th April, with hardly any trace of the complaint.

We would refer our readers to a paper by Dr. O'Beirne, on encysted tumor of the neck, which was noticed in a late Number of this Journal. The scales floating in the watery fluid which Mr. Syme drew off, were no doubt adipocire. They are occasionally present in the water taken, in cases of hydrocele, from the tunica vaginalis.

### III. Ununited Fracture of the Humerus, cured by Excision of the Ends of the Bone.

*Case.* A farmer aged 50, was admitted into Minto-House,* on the 7th of February, on account of an ununited fracture of his right arm. The humerus had been broken two months previously, and he had worn splints for six weeks. Mr. S. found that the bone had been broken very obliquely; the lower end of it lay directly under the skin on the outer side of the biceps, and the upper one was felt quite on the opposite side of the limb, imbedded among the muscles. The limb could be bent in all directions at the fractured part, and the broken extremities were so perfectly moveable, that it was evident no bond of union existed between them. After trying ineffectually the effects of tight bandaging, Mr. Syme resolved to remove the ends of bone.

"On the 24th, an incision about two inches in length was made near the lower end of the bone, which was readily exposed, so as to have the rounded extremity removed by a saw. The other end was then sought for by cutting towards the part where it was felt, from the former wound. The depth of its situation prevented the application of the saw; but the cutting pliers proved sufficient to divide it. A splinter, about two inches in length, was thus detached from the shaft, and as its removal threatened to be attended with difficulty, it was allowed to remain, in the hope that union might be promoted by its presence. Pasteboard splints were applied, with proper bandages, and the patient was confined to bed. Great part of the wound healed by the first intention, a portion of it at the centre only remaining open and discharging a small quantity of matter. The patient suffered hardly any pain, and no constitutional disturbance. The dressing was changed every second day."

On the 8th of March, the patient felt hot and uneasy, and was attacked with intense pain in the left groin and calf of the leg. Soon afterwards the thigh became swollen and painful, and next day the whole limb was enlarged, tense, elastic, and painful when pressed in the course of the femoral vein, where induration was distinctly felt. Vomiting, rapid pulse, hiccup, and the other formidable symptoms that usually accompany phlebitis, were presented, but the patient finally recovered.

"On the 3d of April he put on his clothes for the first time; and on the 12th it was found that the arm had acquired considerable firmness. On the 28th he went home, a distance of twenty miles, the arm being quite rigid, perfectly straight, and not perceptibly shortened. There was still a very slight discharge of matter, but this I understand has since nearly ceased.

There being only three recorded instances of this operation proving successful in ununited fractures of the humerus since the case of Mr. White in 1760, I feel happy in adding another. The cases requiring it must be extremely rare, but when they do occur, it will be satisfactory to know that the cure may be thus accomplished even under circumstances rather unfavourable."

* A sort of Maison-de-Santé.

Mr. Syme expresses surprise at the occurrence of phlebitis of the lower limb in this case, and seems puzzled in what manner to account for it. Had he the experience of a London hospital, his astonishment would speedily subside on the frequent repetition of the circumstance, although the difficulty attending the explanation would not, perhaps, be proportionally lessened. In fact, such a case is far from unfrequent. We have seen inflammation of the veins of the lower extremity at least a dozen, yes more than a dozen times, after injuries and operations in other parts of the body. The phlebitis must be looked on as one of those secondary inflammations that too frequently occur. In the London Hospitals, these cases are almost invariably fatal.

---

### NEWRY DISPENSARY.

### I. DEATH FROM NITRIC ACID POURED INTO THE EAR.

Dr. Morrison, surgeon to the Newry Dispensary, has published this case in the last number of our contemporary of Dublin.*

*Case.* A woman, aged 40, addicted to drunkenness, had a quantity of nitric acid poured into her right ear, on the 6th of June, 1833, while in a state of intoxication. This was done by her husband. She was awoke by the pain, which continued in a slight degree for two or three days, and then subsided altogether. She then however became so weak as to be unable to quit her bed. Six days after the occurrence, a thick slough was detached from the meatus auditorius externus, and this was followed on the next day by a very copious hæmorrhage. On the day after that the woman lost the use of her right arm, and fell into a state of alarming debility. It was on this day, the eighth, that she came under the observation of Dr. Morrison.

There were now several ulcerated spots on the surface of the ear, and the lobe seemed dead. There was a trifling ichorous discharge from the external meatus, and the sense of hearing was lost.—There was no headache, nor were there any febrile symptoms. The following is the curious account of the progress and issue of the case.

" Notwithstanding the plugging of the ear, and the application of astringent lotions, together with the internal administration of tonics, animal broths, &c., the hæmorrhage returned to some extent, almost daily, for about a month, when it ceased, and the general debility during that time increased. A fortnight after the beginning of the illness, the right side of the body, the use of which, from the onset, seemed gradually declining, was so deprived of its usual power, and its different parts so affected with frequent tremulous motions, which even in bed were quite apparent, as to make it evident that one half the body was labouring under paralysis agitans. This latter affection continued about five weeks, when a considerable amendment took place, both as respects it and the general state of the system. The muscles of the right side were now more under the dominion of the will, and the tremulous motions had very nearly subsided. The woman at this time determined on seeing her husband, who was in hospital here, and by the support of two persons, she walked through several streets to him; but on her return home, she felt greatly exhausted, sunk into a state of general prostration, and so continued till her demise, which took place about six weeks afterwards. The side which had been subjected to the paralytic affection, with the exception of the arm, which remained totally deprived of its use, was for several weeks before her death quite free from the tremulous motions, and as capable as the other of the voluntary. Articulation remained distinct, and the mental faculties were unimpaired; the powers of the system at large, more than of individual parts, seeming to have undergone such thorough decay. There was, however, some cough, with muco-purulent expectoration, and night-perspirations."

---

* March, 1836.

*Dissection.* The meatus externus was widened. Opposite the meatus auditorius internus, the dura mater seemed, in a spot of about the dimensions of a sixpence, darker than it should be. At the entrance of the meatus, there was a clot of blood the size of a pea. The right petrous bone was completely carious. No other positive alterations were discoverable.

Dissection has thrown so little light upon this curious case, that it would be idle to indulge in hypothetical considerations. We shall leave the facts as they stand.

### II. Wound of the Epiglottis.

The husband of the preceding patient cut his throat when he saw how matters were going. The wound was inflicted between the os hyoides and the thyroid cartilage. When received into the infirmary the voice was nearly extinct, the cough suffocative, deglutition almost impossible, part of the drink escaping through the wound. Two attempts were made to pass the tube of a stomach-pump down the œsophagus. But they were made in vain, the irritation excited by the attempt being too great to admit of its success. These symptoms gradually subsided, the wound healed, and the patient left the hospital with his health nearly restored, and some cough, hoarseness, and a low croaking voice remaining. Three months afterwards he was re-admitted, to die of low inflammation of the bladder.

On dissection it was found that the epiglottis was deficient in almost the entire of its left lateral half; that its edge at this side was thick and marked by an indentation of about a line in depth; and that the arytenoid cartilage of the same side was considerably enlarged, and as it were forced over towards its fellow, as if to supply the place where the epiglottis was wanting.

Whether the symptoms above-mentioned must be regarded as essentially connected with the lesion of the epiglottis, is little more than conjecture.

---

### Sir Patrick Dun's Hospital.

Dr. Law, physician to this hospital, has lately published some pathological observations on the brain and nervous system,* from which we abstract the following case. Before we mention its particulars, we may notice what we fear are some very just remarks of Dr. Law's.

" No subject of pathology has been more the matter of crude speculation, than the affections of the brain and nervous system. Very few cases have been considered sufficient substratum for a theory promulgated with all the authority of a well-established fact, but whose flimsy pretensions experience was soon to set aside. We could adduce as an example, how premature was the attempt to connect in the constant relation of cause and effect, paralysis of the upper extremity and diseases of the opposite optic thalmus, and paralysis of the lower extremity and diseases of the opposite corpus striatum. Nothing is more injurious to a science, whose conclusions are to be drawn from an accumulation of facts, than a precipitate hurry to generalize, before the facts are sufficiently numerous to furnish adequate data for general deductions; and no science has suffered more from this impatience, and from the over-weaning desire of the eclât of a new discovery than medicine. Many a sickly production has been indebted for its untimely birth and speedy dissolution, to the feverish anxiety to be the first to broach a new doctrine or theory. It must be confessed, that no science has been less fairly dealt with than medicine; none whose records are read with more distrust and suspicion, from the too frequent want of candour and truth with which they are impressed."

The "false facts" of medicine are probably as plentiful as ever. But fortunately, a more rigid censorship is exercised, and scientific mendacity is becoming daily a less profitable trade. It is peculiarly the province of the practical reviewer to separate the wheat and

* Dublin Journal, March, 1836.

chaff. That separation is difficult or easy in proportion to his information, and we too often see the assumptions of impudence, and the inanities of ignorance, pass unobserved and unreproved, in medical societies, and in medical journals. But we proceed to the case.

CASE. *Extensive Cerebral Hæmorrhage—Ossific Depositions in the Arteries —Long-continued Pain in the Head.*

A woman, æt. 43, was admitted into the hospital, with distressing supra-orbital headache; pulse 100, weak; tongue loaded with a dark brown crust at base and in the centre, red and glazed at points and edges; stomach irritable and painful on pressure; no diarrhœa; skin of natural temperature; no depression of spirits. She had been treated for fever by a physician, and Dr. Law, taking the same view of the symptoms, prescribed leeches to the epigastrium, effervescing draughts, &c. But these remedies were of little service, and the Doctor began to think that the essential seat of the disease was in the head.

" What led us to adopt this opinion, was a strength of voice and power of moving herself, apparently incompatible with her other symptoms, as characteristic of low fever. Acting upon this view, we had leeches applied to the left temple, to which she principally referred her pain; she derived temporary relief from their application. We next tried blisters, which also relieved for the time. We now determined to bring the system under the influence of mercury, and for this purpose directed a combination of calomel and James's powder. No sooner had the mouth become sore, than the headach entirely ceased, and the loaded tongue became clean. This complete exemption from pain only lasted so long as the mouth continued sore, for no sooner had this evidence of the mercurial influence passed by, than the pain returned, although much less constant and greatly diminished in intensity. We again resorted to the mercury, which removed the pain never to return; the tongue resumed the loaded appearance which it originally presented."

We would observe—that under any circumstances it would have been proper, we might almost say natural, to have directed the remedies to the head. " Distressing supra-orbital headache" with a brown tongue, and a pulse at 100, are surely symptoms which indicate leeching, cupping, and blistering in the region of the head. But to proceed.

The sight of the left eye now failed, and was lost, the pupil becoming permanently dilated, and complete amaurosis being established. Suddenly she was attacked with total insensibility, laborious breathing, and slow weak pulse. The temporal artery was opened, and ten ounces of blood were abstracted, when the pulse began to fail. Then the bleeding was arrested, and soon the face flushed, and the carotids pulsated strongly. In two hours from the outset of the attack she died.

*Dissection.* The vessels of the scalp, and the superficial vessels of the brain were much congested. About six ounces of fluid blood were found effused at the basis of the latter. A large coagulum occupied the place of the *locus perforatus,* or floor of the third ventricle, which seemed to have been completely destroyed; both crura cerebri seemed to have been elongated and displaced, and apparently had their natural consistence diminished by the violence they had sustained. The lateral ventricles were filled and greatly distended, with two clots of blood which lay in contact, in consequence of the destruction of the septum lucidum. The right optic thalamus was perfectly natural; the left contained a clot in its softened broken down centre. A large coagulum was found in the middle of the great transverse fissure of the brain, inclining rather more towards the right side; this seemed to establish a kind of connexion between the blood effused into the ventricles and that at the base of the brain. The basilar artery was healthy, but the middle arteries of the brain presented many points of ossific disorganization.

Dr. Law remarks that the ramollissement of the left optic thalamus, was probably the original disease which gave rise to the headache and amaurosis of the left eye, for the optic nerves presented no alteration.

Dr. L. directs attention to the state of the tongue, which was brown and loaded, and which had misled the attendant physicians.

"We had seen," he observes, "this condition of the tongue in affections of the head, sufficiently often not to be misled by it; and have been surprised, on pointing it out to more experienced practitioners, to find that it had hitherto escaped their notice. We find Andral, in his lecture on encephalitis, in speaking on the possibility of mistaking this affection for typhoid fever, lays down a clean tongue as one of its distinguishing marks: our experience, on the contrary, leads us to enter our *caveat* not to attach an undeserved value to an appearance of this organ, which so far from helping us out of our difficulty, is calculated to draw us farther into it."

We would observe that whatever may be thought of the abstract nature of fever, it is quite certain that local alterations will occasion all the phenomena of the purest idiopathic fever. We see then no great mystery about this case. The cerebral arteries were diseased, chronic disorganization of the cerebral substance was set up, and low fever was the consequence. This is not unfrequent.

The other cases do not seem to require especial notice. They are instances of hemiplegia.

---

### Clinical Notes of Cases treated in the Westminster Hospital. By John Thurnam, M. R. C. S. Apothecary to the Hospital.

In the following selection of observations from my note-book, I have endeavoured (in a somewhat desultory manner, I must confess), to throw together a few cases, which, either as presenting some feature of rather peculiar interest, or as illustrating some point of pathology or therapeutics, I have thought not to be altogether unworthy of being placed upon record. Should I at any future time venture to lay any further observations before the profession, I trust they will assume more of a *definite and connected* form.

The cases are as follow:—

1. Medullary Sarcoma of the Bones of the Face; operation; death; autopsy.
2. Fæcal Abscess; peculiar changes of the respiratory organs, &c.
3. Extra-uterine Fœtation?
   Diseases of the Nervous System.
4. Hemiplegia with peculiar phenomena.
5. Paralysis from Caries of the 1st and 2nd Cervical Vertebræ.
6. Hydrocephalus Acutus following Scarlatina.
7. Hydrocephalus Chronicus, with Paralysis.
8. Mania; emplastro-endermic mode of administering remedies.
9. Cases of Eczema.

### 1. *Medullary Sarcoma of the Bones of the Face; Operation; Death; Autopsy.*

The disease had existed for about nine months before the admission of the patient (a female aged 46) into the hospital in July last, and commenced with pain in the cheek; tumor of the gum, spreading up the bone, becoming visible externally, and by degrees disfiguring extensively the cheek. The eye was raised by the tumor, as well as encroached on by its greater prominence about the orbital margin; the nose was deflected towards the opposite side: there was a constant discharge of an offensive fluid into the mouth, through the alveoli, from which two of the teeth had been extracted before her admission. The pain became more severe, lancinating, and appearing in paroxysms; and the tumor made rapid progress. Mr. Guthrie soon came to the conclusion that the disease was of a malignant nature, the only remedy for which, (although a doubtful one) was in the knife.

The operation was however deferred on account of repeated attacks of irritability of the alimentary canal, manifesting itself in the shape of diarrhœa and vomiting, which appeared to be induced by the deglutition of the nasty secretion from the walls of the diseased antrum, and which was constantly being poured into the fauces. For this reason, it was not until Oct. 24th, fourteen weeks after her admission, that the operation could be performed. In the course of this period, the tumor had increased considerably, the integuments covering it had become livid, and in one place, where a puncture had been made to give egress to a little matter, had ulcerated.

*Operation.* The tumor was first exposed by making a rhomboidal flap of integument, from the cheek. The anterior prominent part of the tumor, which proved to be the anterior wall of the antrum, was removed with the scalpel, and by this means the posterior wall, exhibiting corresponding marks of disease, was exposed. This was partly done with the view of allowing more room for the succeeding steps of the operation, and partly to satisfy some of Mr. Guthrie's colleagues of the necessity for more deep dissection. As this was now quite apparent, Mr. Guthrie proceeded to isolate the upper jaw from its attachments, by dividing the zygoma, and the ascending orbital process of the jugal bone, (which was involved in the disease) with a chisel and mallet. This was readily effected, from the morbid softness of the osseous tissue in the neighbourhood of the disease. The connexion of the superior maxillary and palatine bones with their fellows was readily overcome in a similar manner, and also that of the nasal process of the former with the frontal and nasal bones. A blunt-pointed knife was employed to divide the lachrymal, and orbital plate of the ethmoid bone. The superior maxillary nerve, where it lies in the spheno-maxillary fossa, having been first carefully divided with the scalpel, the operator endeavoured to raise the diseased mass from its situation by insinuating his fingers beneath it, but it was found to retain some close connexions at its posterior border. These it was necessary to divide with the scalpel, which was only effected with great difficulty from the motions of the tongue.

On its being removed, some of the diseased mass was found remaining in the situation of the amygdala, and the glandular part of the soft palate, and still more towards the ethmoid cells, and sphenoid sinus. This required fresh and careful dissection, and the whole mass was finally thought to be removed. The operation, as far as this point, occupied forty-five minutes, very little blood was lost, not a single ligature was required, and the patient sustained it almost without a murmur. The appearance of the features in this state of vivisection, the ghastliness of the unsupported eye-ball, combined, together with other circumstances better conceived than described, to invest the patient in characters the most appalling that most present had ever beheld.

A combination of the intercepted and the twisted sutures (metallic wire being principally used for the former), was employed in bringing the incised integuments into apposition.

Not a single bad symptom arose after the operation; the ligatures and pins were removed principally on the fourth day, and the remaining ones a day or two subsequently. The patient was for several weeks completely relieved from the severe paroxysms of pain under which she had laboured; her general health improved considerably, and there seemed to be some ground for hoping that the disease would not return.

There was, as might have been expected, considerable difficulty of deglutition, and articulation, but these defects, after a few weeks, began to be less apparent, and the appetite became remarkably good; her strength appeared to be returning, and she was able to take exercise with the assistance of the nurse in the corridors of the hospital.

Unhappily this state of things was not of long continuance. Late in December the characteristic pain which had for some weeks been present in a less degree, became very severe in the forehead, temple, and face;—the cheek which was at first sunken by the removal of the diseased mass, became elevated by the regeneration of the morbid structure; the motions of the lower jaw and of the tongue were impeded, and at last nearly impossible; a tumor gradually appeared in the right temporal region, which ultimately occupied a large space in front of the ear and behind the eye, and extending into the parietal region. As the tumor increased in size the pain became more severe, the general health failed, the appetite becoming less and less, and emaciation becoming more and more apparent.—The pain was relieved considerably from time to time by the application of the extract of belladonna softened with water. She had borne up with amazing fortitude under all her sufferings, but about this period there seemed to be a condition of diminished sensibility of the brain to pain, and the patient lay in a state approaching to stupor. The difficulty of deglutition, pain, and other symptoms continued to increase, and the patient's powers to sink, and on February 12th, about four months after the performance of the operation, she died.

*Inspection of the body four hours after death.* The head was divided vertically in the median line, along with the lower jaw, and the right half was then separated from its connexion to the rest of the vertebral column. In this way the cavities of the head and mouth were examined. The right buccal and nasal cavities, which had become one by the removal of the bones, were filled with the cancerous mass, and the right side of the pharynx and root of the tongue were encroached on by the tumor. The basilary portion of the sphenoid bone, with its sinus, had become the seat of the same disease. The occipital bone was free from contamination. In the anterior cerebral fossa, in the situation of the sphenoidal fissure, the softened mass was perceptible to the touch, but there was no tumor, nor was the dura mater altered. The eyeball, though obscured by the tumefied palpebræ, was nevertheless thrown forwards by the extension of the tumor to the orbit. The disease having its seat probably in the greater and lesser wing of the sphenoid bone. Externally the tumor was seen to be lobulated and of precisely the same characters as that removed by operation, and to have the fibres of the temporal muscle atrophied and stretched over its lower half. The thoracic and abdominal viscera were, with very trivial exception, healthy.

2. *Fæcal Abscess; peculiar Changes in the Respiratory Organs, &c.*

Richard Paton, aged 78, was admitted August 25th, 1835, under the care of Mr. Guthrie. He had a cataract in each eye, and Mr. Guthrie contemplated relieving him by operation.—When he had been in the hospital about a week, he began to complain of pain, of a rather obscure nature, in the abdomen, attended with diarrhœa and tympanitis. This was treated as inflammatory, with leeches, calomel, and opium, mustard cataplasms, &c. When the diarrhœa became more chronic he had chalk and kino mixture, opiate enemata, and latterly, as the powers of the constitution seemed to fail, quinine, brandy, and a nourishing diet were added. About the middle of September, a little swelling and redness were observed in the right groin. A livid colour took the place of the redness. The integuments became thin, a small portion gradually sloughed, and in this way an ulcerated opening was formed which gave egress to an offensive dark-coloured matter, having a strong fæculent odour, and in other respects closely resembling unhealthy fæces. The discharge became more abundant, and was occasionally tinged with blood; the diarrhœa continued, and became more severe; he had occasional fits of rigor amounting to horripilation, with pain in the hypogastric region, of a character still more severe. For these symptoms the starch enema with opium, the hip-bath, and hot fomentations, were employed, with but little advantage.

Early in October a second tumor, having similar characters to the previous one, appeared rather higher up in the groin, which ran a corresponding course, elongated, and discharged a similar matter. The patient gradually became exhausted, ceased to feel pain, the pulse became feeble, and without any fresh symptoms manifesting themselves, he died on the 9th of October.

*Inspection of the body.* The fistulous openings in the groin were found to communicate with a large sloughy abscess in the situation of the iliacus internus and psoas muscles, both of which had become nearly destroyed. The abscess had burrowed between the fascia transversalis and abdominal muscles above the pubes, beyond the median line of the body to the left side, and likewise extended downwards into the pelvis, by the side of the rectum, to about half an inch or so above the anus. There was no carious disease of the vertebræ or iliac bones.

The smaller, and last-formed fistulous opening in the groin, passed between the external iliac artery and vein, to communicate with the abscess. The proper tunic of the latter was very thick, like that of an artery, but at the point where the sinus was in the closest contact with it, inflammation had set up in its interior, its canal was partially obstructed by coagulated lymph, the internal and middle coats were ulcerated for the space of about an inch, the continuity of the canal being, for this extent, preserved merely by the thin external cellular coat. The other sinus passed under Poupart's ligament, externally to the iliac vessels, to communicate with the abscess. The intestinal canal communicated with the abscess at three points. First, a knuckle of the ileum had become adherent to the peritoneum covering the iliac fossa, at about ten inches from the termination of the small in the large intestine, and here an opening as large as the point of the forefinger existed. There was a still larger communication with the upper and inner part of the cæcum, and one, smaller than either of the others, with the colon about two inches above the cæcum.

It will be observed, that the point where the opening into the cæcum existed, was precisely that which in a healthy condition of parts is occupied by the mouth of the appendix vermiformis cæci. Search was in vain made for this little organ in the peritoneal cavity, and it was only on very close examination that it was found extending from the opening described in the cæcum into the cavity of the abscess, with the parietes of which it had become adherent. It was pervious, but highly thickened, and at its extremity disorganized. There was scarcely a trace of the ileo-cæcal valve of Bauhin, and the opening of the ileum into the cæcum would scarcely admit of the point of the little finger. The mucous membrane of the large intestine was in a state of livid congestion, as well as of ramollissement; there was one large and distinct patch of ulceration about the commencement of the colon: a large clot of blood was found in the rectum. The abscess just below the cæcum was only intercepted from the serous sac of the abdomen by the peritoneum itself, which during the dissection was ruptured by an exceedingly slight force.

*Thorax.* The lungs were crepitant, had traces of carbonaceous matter here and there on their surface, but were in a rather exsanguine and emphysematous state. The heart was, if anything, rather under the medium size, and had a white spot on its surface rather larger than a shilling. There might be a little dilatation of the right cavities. The left ventricular cavity was contracted, from very considerable concentric hypertrophy of its walls, so that on dividing it, it would scarcely admit the forefinger. The walls were one inch, and in some places a line or two more, in thickness. The auriculo-ventricular opening was patescent from the existence of a rather considerable ossific deposit in the base of the mitral valve, occupying two-thirds of its extent.—There was a little dilatation of the left auricle, attended with some hypertrophy, chiefly of the musculi pectinati. The substance of the liver was healthy, but presented three remarkable depressions in the shape of sulci, on its convex surface. These sulci were found to correspond with a similar number of highly hypertrophied muscular fasciculi of the diaphragm; and a like state of this muscle existed also on the left side. The bodies of many of the dorsal and lumbar vertebræ, as well as their connecting fibro-cartilages, presented numerous irregularities, from the presence of exostotic deposits.

That the disease which proved fatal in this instance, had its origin in some obstruction in the mouth of the vermiform appendix, giving rise to inflammation, ulceration, and consequent abscess, an attentive consideration of the morbid appearances would, I think, lead us to conclude. Another sentiment, however, was expressed by some at the examination of the body, viz. that the disease had originated in the muscles and had only implicated the intestine secondarily. The following considerations would, I think, tend to establish the view of the case which I advocate, and which I believe to be applicable to most of the cases of this description which have been published. Cases of abscess in the right iliac fossa, involving the cæcum, and generally leading to fistulous openings in the abdominal parietes, are not very unfrequent, and have been recorded by Dupuytren, Dr. Copland, and others; but did they *commence* in the external parts, and were they *primarily* inflammations of the "pericæcal tissue," to use the term given by Dr. Copland to these cases? Can any reason be alleged why they should not be of equally frequent occurrence in the left iliac region? and that this is the case will not, I suppose, be contended.

Paton's case is also interesting, from the appearances met with in the respiratory organs. The fasciculated diaphragm, fissured liver, semi-anchylosed and exostotic vertebræ are appearances, coupled with a peculiar condition of the tissue of the lungs, and of the bony walls of the thorax, which, it seems, from "Clinical Researches on the Diseases of Old People," recently "conducted at the Hospital of the Salpetrière, by MM. Hourman and Delambre," are extremely common in the bodies of persons in advanced age.*

3. *Extra-Uterine Fœtation?*

Sarah Bailey, aged 30, was admitted, Oct. 27th, under the care of Dr. Bright. She sought admittance into the hospital more on account of a bronchitic affection, than of the circumstances which are here to be detailed. She gives the following history of her case:—During the last fifteen years, has lived in a state of concubinage with a sailor; has had no children; miscarried seven years ago. Five years since she became pregnant, "quickened about the middle of her time," was very large, but continued to menstruate during the whole nine months, and, with the exception of the last four months, during which time the catamenia have been rather irregular, has continued to do so up to the present moment. At the expiration of the nine months, pains, supposed to be those of labour, came on, which for some days were very severe—but at the end of a week left her. The abdominal tumor has remained ever since, though a good deal less than during the nine months.

At the present time, there is a distinct tumor in the umbilical and left hypochondriac regions, which is peculiarly prominent and distinct when she lies on the left side, and is terminated in a roundish protuberance, about as large as an orange, just under the cartilages of the false ribs. A secretion of watery milk took place in the left breast at the time, but this has given place to a very remarkable blackish-green secretion, which she can at any time express from the nipple.

### Diseases of the Nervous System.

4. *Hemiplegia, with peculiar Phenomena.*

The case of Francis Armstrong, a patient of Dr. Roe's, may be perhaps worthy of notice, as an example of apoplectic seizure, followed by hemiplegia at an early age, viz. 23, without any ade-

* Since the above was written, I have been fortunate enough to have had two or three other opportunities of confirming the interesting observations of these gentlemen, who I am happy to see are continuing their researches. It would swell this communication to an unreasonable length, to introduce the particulars of these cases here.

quate cause that can be assigned for its production. Recovery of power in the limbs is taking place gradually; the arm was nearly stationary during a fortnight, but at the present time, seven weeks after the seizure, is going on satisfactorily, under the repeated application of blisters over the anterior wall of the axilla. Before the blister was applied, he noticed that, although the muscles of the arm were entirely insensible to the stimulus of volition, yet that, in the actions of gaping or sneezing, the arm was involuntarily brought into a state of flexion. I directed his particular attention to this symptom, and, from the uniformly-consistent nature of his statements, I can place confidence in them. The motions were confined to the fore-arm.

The interesting nature of the bearing which this fact has on Sir Chas. Bell's theory of a respiratory system of nerves, will at once be seen. Setting aside the theory, however, or rather hypothesis, as I believe we must at present deem it, the case fairly establishes the close bond of sympathy which exists between the ordinary muscles of respiration, and those of the extremities; and that to an extent even greater than Sir Charles, as far as I recollect, has himself shewn.

5. *Paralysis from Caries of the 1st and 2d Cervical Vertebræ—Autopsy.*

Anne Josey, aged 16, was admitted August 22d, into Percy Ward, under the care of Mr. Guthrie. The complaint arose nine months ago, after exposure to a draught of cold air, in the shape of a painful stiff neck, which at first affected the right side, but, after some time, became most severe on the left. This gradually got worse up to the period of her admission.

Sept. 4th. There is great pain in the neck, with swelling, especially on the left side and towards the nape. The head is bent downwards and towards the left side, the face looking to the right, apparently in consequence of the contraction of the sterno-mastoid and other muscles, supplied by the spinal-accessory nerve. There is paralysis of the upper extremities of both sides, both as regards motion and sensation. The paralysis does not appear to extend to the lower extremities, nor is there any loss of power in the sphincter. The urine is sometimes retained, requiring the use of the catheter; there is also a good deal of diarrhœa. The muscles of the face and jaws are the subject of a painful spasmodic affection, resembling trismus. Eyes prominent, pupils dilated—anorexia—tongue red—pulse accelerated, with other febrile symptoms

The left arm was first affected with the paralysis—it is now œdematous, and its surface presents several round and oval, elevated and circumscribed spots, some of them as large as a half-penny. They have a reddish and inflamed base, and would seem to consist of an elevation of the cuticle, by the presence of lymph in the areolæ of the so-called rete mucosum of Malpighi. The patient gradually became worse, and died on the 6th September.

The autopsy revealed the existence of an abscess behind the pharynx, containing healthy pus, and involving the substance of the longi colli muscles. It was lined by a very thick velvety membrane, and extended from the base of the skull to the fourth cervical vertebra. The anterior arch of the atlas, and the body of the vertebra dentata, were in a carious state, and the odontoid process was broken off. The membranes of the spinal cord, opposite the diseased bones, did not present any unusual vascularity, but were slightly thickened. The spinal cord was somewhat compressed, and was darker in colour than natural at this spot. The brain was healthy.

6. *Hydrocephalus Acutus, following Scarlatina.*

This case was remarkable in being a sequel of scarlatina, occurring in connexion with the ordinary exanthematous dropsy. The patient, a servant-boy, aged 16, was short-necked, and rather plethoric. At the time of his admission (Oct. 13th), he became convalescent of scarlatina, a fortnight ago, for the last week of which, however, he had been suffering from general dropsy. There had been no head symptoms up to the day of his admission. On the morning of that day, he awoke with severe pain in the head, especially in the temples, and a symptomatic amaurosis disabled him from dressing himself. He was seized with a convulsive fit whilst waiting in the out-patient's room, and, upon his removal to the ward, these were repeated with great severity and frequency. The dejections and urine were expelled involuntarily during the paroxysms. At other times he remained insensible, was very restless, and constantly moaning, tossing his head about, &c. A more than slight febrile action disturbed the regularity of all the functions. He was bled to 16 ounces—the head was shaved, and a cold evaporating lotion constantly applied. During the night he had several convulsive fits. On the morning of the 14th, he lay in a completely insensible state grinding his teeth and moaning; the eyelids constantly closed, pupils contracted. There appeared to be a slight hemiplegiac state, the right leg not responding so readily to stimuli as the left, and there being a slight distortion of the features to the left side. At 11, a.m. ℥x. of blood were taken from the nape of the neck by cupping, and he was likewise ordered a saline diaphoretic mixture.

At 4, p.m. he was found much better; has only had two short fits since he was cupped, and he is now able to answer questions. The grinding of the teeth is gone, and he has a good deal of quiet repose.

On the 15th, he had regained complete vision; the convulsive fits, of which he only had two in the course of the day, were very slight. From this time he continued to improve rapidly, and, on the 27th, was dismissed as cured. There was, however, as I thought, a slight hemiplegiac condition—the right arm appeared to hang somewhat more motionless by the side than the other, and the foot of the same side seemed to drag a little.

7. *Hydrocephalus Chronicus, with Paralysis.*

Hannah Gough, aged 20, having suffered from chronic hydrocephalus since her infancy, was admitted Nov. 3d, under the care of Dr. Roe. Her friends, however, it would appear, obtained her admission, more with a view of obtaining relief for a chronic eczema of the scalp, neck, and axilla, under which she has laboured during the last 18 months, than on account of the hydrocephalic condition. The eczema was developed six months after the healing of a seton that had been inserted in the nape of the neck, when a patient at Guy's Hospital; and to this cause it has been attributed.

Under the employment of the vapour-bath, and of a mild alterative, she is getting rid of the cutaneous disease. The case is a well-marked one of chronic hydrocephalus. The head measures fifteen inches from the nasal process of the frontal bone to the occipital tuberosity—and the same, arching over the cranium, from one mastoid process to the other. It is twenty-two inches in circumference, taking the line usually described in sawing through the skull, for the purpose of removing the calvarium.

There has been partial paralysis of the right arm during the last three years; it now principally affects the extensors of the hand, as in the case of "dropped hand" from lead. The hand of this side is pale, but of good temperature, and the pulse at the wrist is strong and full. The contrast between the two hands is very striking. There is no actual paralysis of this side, but for some time she has experienced a painful degree of numbness in the arm. This is attended with coldness; the hand has now a puffy tumidity about it, has a reddish and rather livid colour, feels cold and clammy to the touch—the skin is rough and harsh; and the tout-ensemble is very much that of a hand after long exposure to a freezing atmosphere. The pulse at the wrist is 96, as on the other side; but it is feeble, soft, and much more readily compressed.

8. *Mania; Emplastro-Endermic Method of Medication.*

The case of mania was very acute and rapid in its progress to a fatal termination. The subject of it was a female, aged 20, of respectable connexions. The case had been regarded as one of delirium tremens, and she was brought to the hospital two days after the first attack. It was difficult to obtain any correct history of her habits, &c. as she had been living in a secluded manner. Anxiety on pecuniary matters, excitement on the subject of religion, and the softer passions were all adduced by different of her acquaintance—some alledging one, and some another. It is not improbable that all these causes had some share in the development of the disease. The madness was of the most furious kind, requiring constant restraint; when she first came in, religious subjects of the most melancholy description were the burden of her ravings, but, towards the latter days of her life, sexual ideas seemed to predominate. Little or nothing in the way of food or medicine could be got down, and, on the 9th day of the disease, she died, probably as much from the effect of inanition, as from the exhaustion produced by the disease. The case is related here principally for the purpose of alluding to the advantage of the "emplastro-endermic" mode of administering remedies in mania. In the present instance, a blister had been applied to the nape of the neck, and I took advantage of it for this purpose. One grain of muriate of morphia (afterwards increased to 2 grains), rubbed up with a few grains of lard, was applied to the blistered surface at bed-time. The happy effects of stealing the remedy into the system in this way, manifested themselves, on the first application, in procuring a tranquil state—and, on the second, in obtaining for her, for the first time, several hours' sleep.

The inspection of the body after death did not throw any new light on the pathology of mania. There might, perhaps, be a somewhat anormal degree of vascular congestion, both in the cerebral substance and its proper meninx. There was a slight degree of simple hypertrophy of the left ventricle of the heart; and the internal genital organs were in a high degree of congestion.

9. *Eczema.*

Of the cases of cutaneous disease which have presented themselves at the hospital, those of eczema have been the most interesting. Three cases, during the last eight months have been observed, in which no local cause could be assigned for the production of severe and extensive eczema of the impetiginous variety, which in two became chronic; nor yet any constitutional one, except a deranged state of the uterine function. Two of these females were suffering uterine disturbance at the "critical epoch," and the third was pregnant. This patient died in the hospital from puerperal fever, following abortion about the fourth month of utero-gestation. The patient aborted almost immediately on the disappearance of the eruption, after a small blood-letting. She had, however, been taking likewise the expressed juice of the menyanthes trifoliata. What share had this remedy in the production of the abortion? A highly interesting case of what seems to be modified eczema rubrum, having a recurrent and highly inveterate form, appears to merit a detailed notice, especially as having been communicated to the Westminster Medical Society, it has since been published in an imperfect and mutilated form.

10. *Modified and inveterate Eczema-rubrum.*

Thomas Cass, aged 24, a carpenter, was admitted Nov. 17th, 1835, under the care of Mr. White.

At the period of his admission the following particulars were noted. Tall and thin in stature, of pale complexion, and deeply marked with the cicatrices of small-pox pustules. The surface of the body in many places presents the cuticle in a state of desquamation, especially that of the thighs, the mammæ, and the plantar surface of the feet. In the latter situation the process is in its least advanced stage. The palmar surfaces of the hands and fingers have only recently parted with their cuticle, are extremely red and tender, and are studded with numerous minute drops of perspired fluid. The appetite is good; bowels regular; tongue reddish, slightly furred in the centre; pulse 84, very full, of medium strength; complains of a feeling of general uneasiness in the chest, accompanied with sensations of "rumbling and fluttering;" impulse of the heart somewhat increased.

*History.* Six years since had small-pox very severely after vaccination, the left eye becoming nearly useless from extensive corneal opacity. After this he had good health during four years. At this period he was for some time employed at Jones's Match Manufactory at Battersea. A month or so after this, and two years ago, an eruption appeared all over the body of minute vesicles, or as the patient termed them "pustules." These were about the size of a pin's head, the intermediate portion of the tegument being of a deep red colour, the development of the erythema having however been prior to that of the vesicles, by a day or two, and before the latter appeared he had taken powerful diaphoretics. There was burning heat, severe pain, and excessive pricking and itching of the skin. In the course of a short time, the vesicles ran into each other, and were ruptured, either spontaneously or by the patient's scratching them, and they discharged a watery fluid. The entire course of the attack appears to have occupied about six weeks, and its termination to have been in a moist desquamation of the cuticle;—the "dark squameuse humide" of Alibert. Preceding and accompanying the local affection, there was considerable disturbance of the functions; viz. pain in the head, dazzling light before the eyes, slight delirium, uneasiness in the chest, with a painful feeling of oppression and beating all over it; tongue very white, rough and furred, appetite but little affected, no thirst, urine scanty and high coloured, no sore throat. Twelve months after this he had a second attack, having precisely the same symptoms, course, and termination. Six months had scarcely elapsed, when, five months before his admission, he had a third attack, differing from the preceding ones in the absence of all vesication, and consisting merely in an uniformly diffused, and extremely deepred blush. He had not taken as before any diaphoretic remedies. He has since had two attacks without vesication; these have followed each other at intervals of about six weeks; and terminated in the entire desquamation of the cuticle, in a dry state. It is the last and fifth of these attacks, the last stage of which he now labours under. He has not taken mercury in any form. Whether this mineral enters into the composition of any of the matches, made by Jones and Co. at Battersea, is not known.

Nov. 22nd. He went to bed as well as usual; about 2 A. M. he awoke complaining of great heat of surface, and had a rigor which lasted more than an hour, with great pain in the head. A deep red rash appeared on the neck, which before morning had spread all over the body. In the morning the entire surface was of a deep brownish red colour, without tumefaction or vesicles, the skin dry, tongue moist, furred, white; pulse 108, full.

*V. S. E. B. ad* ℥xiv.

℞. *Mist. Diaphoret.* c̄ *Antim. Tart. capt.* ℥iss. *ter die.*

℞. *Haust. Cathart. p. r. n. sumend.*

23rd. No sleep, urine scanty, bowels open, skin dry and hot, of a deep coppery red. To-day, for the first time, a few flat and irregularly-shaped confluent vesicles were noticed between the shoulders. These contained a little yellowish serum.

*V.S. E.B, ad* ℥xij.

℞. *Mist. Effervescens.* c̄ *Sp. æth. nit. capt.* ℥i. 2*dis. horis.*

24th. The cuticle has already commenced desquamating, especially about face and shoulders; the surface generally is less red and hot; bowels open, tongue less furred, head-affection almost gone, urine more abundant.

25th. The general symptoms are all much relieved, except that he still complains of pain in the head. Very extensive desquamation of the cuticle is going on, large laminæ peeling off in all directions. The arms and upper part of the body are already nearly clean. The epidermis of the hands appears likely to separate in the form of complete cuticular gloves, the chirothecæ of the older anatomists.

*Persistat in usu medicament.*

26th. This morning, on visiting the ward, found his bed full of pieces of exfoliated epidermis, which was also strewed by the bedside. Entire desquamation has now nearly taken place. Two layers of cuticle, however, on the plantar surface of the feet, that separated by the preceding and the present attack, remain to be exfoliated. The gloves of cuticle alluded to in yesterday's report, have been removed entire from the hands; in order to do this, it was found necessary to slit up the cuticle covering the backs of the fingers, and to cut it around the basis of the nails. This evening he has severe pain of the head, which was relieved by a fit of epistaxis.

28th. Cuticle almost entirely separated from the whole surface. Tongue less red; urine abundant, pale; still complains of extreme throbbing and pulsation all over the body, but especially in the head, where he can count the pulse—96, full and soft.

Dec. 3d. The new cuticle on the palms of the hands presents numerous dry cracks and fissures, and to the touch of the observer feels like a piece of dry crackling parchment; complains of pain and heat between the shoulders.

6th. The cuticle is again separating, in very fine, almost pulverulent pieces, without any preceding vesication or erythema. During the last week, he has been taking the following mixture prescribed by Mr. White.

℞. *Quiniæ sulphatis*, gr. xvj. *Liq. arsenicalis*, ♏ xlviij. *Pulveris acaciæ*, ʒ ij. *Aquæ*, ℥ viij. *Misce fiat mistura. Cap. ter quotidie.*

This has however been relinquished for the antiphlogistic plan, combining with it the use of diaphoretics and diuretics. *V.S. E.B. ad* ℥xij.

26th. Had an attack of the erythema without any vesicle.

27th. During the last week the cuticle again desquamated as the result of the attack reported on the 28th. This morning another attack of the modified eczema has appeared all over the body; the eruption, as on former occasions, being of a coppery red colour, attended by heat and tingling. Dr. Addison, who kindly saw the case this morning, thought that with the aid of a lens he could detect what he regarded as abortive and imperfect vesicles, in the form of very minute elevations of the cuticle not containing any appreciable fluid. Tongue red; no tenderness on pressure in the epigastrium, or other symptoms of gastro-enteritis.

Feb. 6th. Since last report he has, with very little exception, had an attack, slight and partial only, of the eruption every Sunday morning, which has been followed by but very trifling exfoliation of the cuticle. No very decided plan of treatment has been followed during this interval, and the case may be said to have been treated on the "methode expectante." This morning, however, the surface is covered by a more severe, and altogether general erythema, of the usual coppery-red hue, (without any vesicles,) and attended by the accompanying heat and pain, and a slight febrile movement. The antiphlogistic plan with colchicum has been adopted.

15th. The cuticle has exfoliated universally;—he has continued to complain of the great throbbing of the subcutaneous capillaries, beating in the head, and palpitation or "faultering" of the heart;—to use his own language, "it seems as if the blood were in a ferment." There continues to be rather more redness of the tongue than is normal, especially at its sides and tip, and likewise of the pharynx and soft palate.—*Fiat venesectio ad* ℥vj. *semel in hebdom.*

℞. *Acidi hydrocyanici*, ♏ xvj. *Acidi tartarici*, ℈ iv. *Aquæ*, ℥viij.—*Misce. Capt.* ℥j. *ter die, cum* ℥j. *Mist. sequent. in impetu effervescentiæ.*

℞. *Sodæ carbonatis*, ʒij. *Aquæ*, ℥viij. *Misce.*

*Habt. balneum calidum omni nocte. Serum lactis bovin* Oj. *quotidie.*

Low diet with a mutton-chop. To take daily exercise in the open air. The above plan of treatment has been adopted, at the suggestion of Dr. A. T. Thomson, who saw the case yesterday, and regards the cutaneous affection as symptomatic of some gastro-enteric irritation; as however the treatment seemed equally appropriate with another view of the essential pathological condition, viz. an increased and disordered activity of the arterial system; the plan was cheerfully entered into by those who differed from Dr. Thomson in his view of the case.

Feb. 25th. Since last report some amendment appears to have taken place; there has been no return of the eruption for nearly three weeks; less heat and tingling of the surface, &c. &c. The treatment adopted on the 15th is still continued.

---

### Clinical Report on "Hydrocele of the Neck." By Mr. Bransby Cooper.*

Our readers are aware that Dr. O'Beirne, of Dublin, has lately drawn attention to the formation of serous cysts in the neck. Hydrocele of the neck has been the term applied to the affection. Mr. Bransby Cooper has published a case of this description in the Guy's Hospital Reports. It admits of a good deal of abbreviation, and the following are its principal features.

*Case.* A private in the Royal Horse Artillery, aged 20, was admitted into Guy's Hospital, on the 3d of June, 1835, with a tumor situated under the left sterno-mastoid muscle; reaching posteriorly, so as to occupy the whole space between it and the trapezius: in its vertical axis, it extended from just below the mastoid process to the junction of the middle with the scapular third of the clavicle, and horizontally from this point to the sterno-clavicular articulation. The lower and anterior part of the tumor was the most prominent, and of the most recent formation: the whole swelling seemed as if divided into three distinct lobes; and although they each conveyed the sensation of fluctuation, their cysts did not communicate. The tumor moved with the larynx in the act of deglutition, but appeared to be independent of the thyroid gland. The integuments were of a natural colour, and the veins of the neck were not swollen. The tumor had first been observed twelve months before his entrance into Guy's Hospital, and had gradually increased. Iodine had been more than once exhibited, but although it did no good to the tumor, it did harm to the general health. The man was therefore discharged from the army.

"On the 10th of June I made a small incision down to the sac, at the most prominent point of the anterior cyst, and punctured it with a lancet; when about five ounces of a serous fluid escaped, the first portion of which was clear, but about the last two ounces were coloured with blood. I then introduced a long canula into the cyst, furnished with a trochar, to which were attached four threads of silk; and pushing the trochar through the skin on the opposite side, thus passed a seton through the cyst. The tumor was then diminished about one-fourth in size, shewing that one cyst only had been opened; but I thought it more prudent to await the result of this operation, than to evacuate the three cysts at one time."

On the 13th, pyrexia occurred, with much pain and swelling of the cervical glands. In the evening these symptoms had become aggravated, there was much difficulty of respiration and of swallowing, the tonsils were enlarged, and an unhealthy serous discharge issued by the side of the seton. The latter was accordingly removed, and leeches, calomel and antimony, &c. prescribed. The febrile and inflammatory symptoms subsided, and on the 17th, the wound made by the seton, having ceased to discharge, a female catheter was introduced into the cyst, and about an ounce of sanious fluid let out. This operation was repeated on the 18th. The tumor was then not above one fourth of its original size, and it appeared as if the other cysts had ul-

* Guy's Hospital Reports.

cerated and evacuated their contents. On the 19th very little fluid escaped, upon the introduction of the catheter; but upon pressure at the posterior part of the tumor, in the situation of one of the cysts which had not been opened by the seton, nearly half an ounce of less bloody fluid issued.

On the 1st of July, Mr. Cooper directed some pressure to be made with strips of the emplast. ammoniaci c̄ hydrargyro. This was done for about a fortnight, when the tumor was reduced to an eighth of its original dimensions. Then again was the patient attacked with fever, difficulty of swallowing, inflammation of the throat, and all the former train of symptoms, and again he required leeches, calomel, &c.

" On the 24th, on examining the tumor, it was found to have increased considerably; and a distinct fluctuation could be perceived at its posterior and inferior part, which, on pressure, forced the fluid towards the cicatrix of the opening made by the seton, proving the existence of a sinus across the transverse dimensions of the tumor. I therefore made a counter-opening; and discharged about two ounces of clear serum; and introduced a small piece of lint into the opening."

After this the discharge became bloody, and flakes of adhesive matter were discharged from the new opening, with much constitutional irritation. The tumor, however, grew smaller, the bad symptoms disappeared, the counter-opening healed, and nothing of moment happened till the 5th of September, when a small abscess formed near the opening originally made by the seton. It was opened, and a little inflammation ensued. On the 1st of October, a small portion of the tumor again secreted, and led to the necessity of another opening being made; and about three drachms of serum were discharged:—a piece of lint was introduced into this small cyst, which was followed by some slight degree of constitutional irritation, but this subsided directly the lint was removed; the opening quickly healed, the cyst became obliterated, and, after this, he had no further relapse. He was kept in the hospital till November, when he was discharged cured, rigidity of the neck, from the contraction and obliteration of the cavities of the cysts, alone remaining.

" I once admitted into Guy's Hospital," says Mr. Cooper, " a patient—who was sent up to me by Mr. Rump of Holt in Norfolk—who was the subject of deep-seated tumor on the left side of the neck. which produced difficulty both in breathing and swallowing. From the manner in which it was tied down by the muscles, fluctuation was but very indistinct: and I had the patient brought into the operating theatre, with the idea that I was about to remove a solid tumor: but in the progress of a careful dissection for its excision, the sac was opened, and several ounces of fluid escaped; thus proving the true nature of the case. I introduced a portion of lint into the sac; which became obliterated partly by adhesive inflammation, and partly by the process of granulation. The patient was discharged perfectly cured.—This case I consider to have been completely of the same nature as the last described; namely, an adventitious formation of a serous cyst, and which is certainly not inappropriately termed a Hydrocele.

I believe the introduction of a seton is a better mode of treatment than injection, in these cases; as it affords a more ready means of removing the source of irritation, and is less protracted than the employment of caustic for the purpose of producing obliteration."

Our readers will perceive that, a few pages back, in a Report from the Royal Infirmary of Edinburgh, is a case of " hydrocele of the neck," cured by the seton. The cases which have lately been published, and most, if not all of which, have been collected in this Journal, establish the not unfrequent occurrence of the disease, and the general success that attends the employment of the seton. At the same time, this must not be supposed to be quite unaccompanied with danger. The case of Mr. Cooper exhibits severe symptoms from its use; and the fatal results that have occasionally followed the measure, in cases of serous cysts in other parts of the body, should make the surgeon chary of officious meddling with irritable constitutions.

---

### Clinical Report on Aneurysm.*

### 1. Case of Femoral Aneurysm;—External Iliac Artery tied by Sir A. Cooper. Dissection of the Limb at the end of eighteen years.

A man, aged 39, was admitted into Guy's Hospital, June 22d, 1808, on account of aneurysm of the right femoral artery, immediately below the ligament of Poupart, which was raised considerably by the tumor. This was as large as a small pint bowl, and the skin over it was thin, tense, and purple. The disease had appeared in the Spring of that year, after a walk of five miles with a heavy burthen.

The operation of tying the external iliac, a little above the ligament of Poupart, was performed on the day of his admission by Sir Astley Cooper. Two ligatures were applied, and the artery was divided between them.

We need not pursue the details of the case. It is enough to observe that a good deal of suppuration about the wound and sac ensued, and the patient did not recover for some months from these immediate effects of the operation. He lived, however, for eighteen years and a half after it, and died in the Autumn of 1826.

The limb was injected from the common iliac artery, and, a careful dissection being made, it was put up as a preparation in the museum of the hospital, where it now is. The following is the account of the mode in which the circulation was established and carried on. It is drawn up by a very good anatomist, Mr. Cock.

The description is methodically addressed to these three points. 1. The condition of the iliac trunk above the site of the ligature. 2. The appearances presented by the vessel immediately below the ligature; together with the situation and remains of the aneurysmal tumor, and the femoral artery as restored by the influx of blood from anastomosing branches both above and below the sac. 3dly. The manner in which the restoration of the femoral trunk had been effected by collateral circulation.

*Condition of the External Iliac Artery.*

The external iliac artery was pervious, to the extent of rather more than an inch from the bifurcation of the common iliac; but had become somewhat diminished in size, and altered in shape. No branches were given off from this portion of the vessel, which, when filled with injection, presented a conical form, tapering downwards to a mere point, and terminating in a rounded cord, which constituted the remaining part, or the obliterated portion of the artery, and was continued down to the spot where the operation had been performed. The ligature had probably been applied just above the origin of the circumflex and epigastric branches, although no evidence remained to indicate the precise spot. Just above Poupart's ligament, the iliac trunk became suddenly restored (apparently by the influx of blood from the branches mentioned above), and assumed about half its natural size. The obliterated vessel presented the appearance of a continuous unbroken cord, from the cessation of the iliac above, to its restoration below.

*Condition of the Iliac and Femoral Artery below the Ligature.*

It has already been stated, that the iliac trunk became restored just above Poupart's ligament, by the retrograde circulation established through the circumflex and epigastric branches, which received their blood from the branches of the internal iliac above. The vessel having thus regained about half its natural size, passed into the thigh, and was continued without receiving any accession from collateral vessels, until it reached the origin of the profunda. From this branch (*i. e.* the profunda)

* Guy's Hospital Reports.

the trunk appeared to derive a large supply of blood, sufficient to restore it to the ordinary extent of calibre which the femoral possesses in a stout muscular limb; the remaining portion of the femoral artery, below the profunda, presented nothing unusual in its appearance, and bore no indication of having received any further influx of blood through collateral branches. Just above the origin of the profunda, the femoral artery had become distorted, and irregular in shape; and was rendered somewhat obscure by its connexion with what appeared to be the remains of the aneurysmal sac, adhering to the anterior surface of the vessel, and gluing it to the adjacent muscles and fascia. There can be but little doubt, that the original opening of communication between the sac and the femoral trunk had existed at this spot, viz. just above the profunda branch; but it would seem equally apparent, that, as the aneurysmal tumor became obliterated in the progress of the cure after the operation, the opening into the vessel also became closed, while the integrity of the arterial trunk, above and below the sac, was maintained continuous and entire.

*The manner in which the Restoration of the Femoral Trunk had been effected by the Collateral Circulation.*

The collateral circulation had, in this instance, been established by the junction of the ileo-lumbar, obturator, gluteal and ischiatic branches, from the inter-iliac, with the circumflex and epigastric of the external iliac, and the profunda of the femoral. They consisted of three sets of communicating vessels, which descended, respectively, over the fore part, the internal side, and the posterior surface of the hip-joint; and may be described as forming a vascular plexus around the articulation, ramifying amongst the muscles of that region.

The anterior set consisted—1. Of a very large branch from the ileo-lumbar artery, which descended along the crista of the ilium, to terminate in the circumflexa ilii and circumflexa ilii externa. 2. Of another branch from the ileo-lumbar, which became joined by a small artery from the obturator, and divided into a number of small and exceedingly tortuous vessels. These connected themselves to the anterior crural nerve; and, descending under Poupart's ligament, terminated in the external circumflex branch of the profunda. 3. Of two other branches from the obturator, which turned over the brim of the pelvis, and formed a plexus similar to the last, which, after communicating with the epigastric, descended on the inner side of the femoral trunk, and terminated in the internal circumflex branch of the profunda.

The internal set consisted of the branches, given off by the obturator after it had left the pelvis, which ramified amongst the adductor muscles on the inner side of the hip-joint, and joined most freely with the divisions of the internal circumflex from the profunda.

The posterior set of anastomosing vessels was constituted—1. By three large branches from the gluteal; two of which crossed the dorsum of the ilium, in close contact with the bone, and anastomosed near the anterior and superior spinous process, with the ascending branches of the external circumflex; whilst the third descended in a direction more nearly vertical, between the gluteus maximus and medius muscles, to join with the middle branches of the same artery, just below and behind the trochanter major. 2. By several slender and very tortuous vessels from the ischiatic which surrounded the great nerve of that name, and thus, descending behind the thigh, communicated with the internal and external circumflex, and, finally, terminated by joining the perforating branches of the profunda.

In this preparation, the ileo-lumbar, obturator, gluteal and ischiatic arteries, will be seen enormously dilated. The internal pudic is also of large size; but it does not appear to furnish any direct communication with the femoral.—Doubtless, however, in the dissection of the limb, many small branches were removed, which, during the life of the individual, lent their tributary streams to swell the femoral trunk, and assisted in restoring to the limb that supply of blood which enabled it to maintain its muscular development, and carry on its functions with undiminished vigour.

This is a very interesting dissection, and may be justly added to the stock of valuable facts, connected with the operation for the cure of aneurysm. We are delighted to see one of the early cases in which that operation was performed, placed permanently on record, as another of the memorials of days gone by, and of surgeons whom the profession will always regard with respect, and with affection. We are glad that our old friend, Sir Astley, has communicated the details of one of his early triumphs to the public.

II. First successful Case of Ligature of the Common Carotid Artery for Aneurysm; with the Dissection, Thirteen Years afterwards. By Sir Astley Cooper, Bart.

Again we have before us one of the earlier exploits of Sir Astley. The patient, whose case this is, was a man aged 50, who had an aneurysmal tumor, apparently of the internal carotid, situated just below the angle of the jaw, on the left side of the neck. The operation of tying the common carotid artery was performed by Sir Astley on the 22d of June, 1808, in the theatre of Guy's Hospital. No symptoms of any consequence succeeded the operation; the ligatures (two were used, and the artery was divided between them) separated on the 13th and 14th of July; on the 17th, the patient walked out of his ward; and, in September, he was discharged from the hospital, his recovery having been retarded by a sinus which remained in the track of the ligature. In 1821, the patient died of an attack of apoplexy, and the body was examined by Sir Astley Cooper and Mr. Key.

*Dissection.* An injecting-pipe was introduced into the arch of the aorta, and common coarse injection thrown in, the subclavian arteries being previously tied external to the scaleni.

The head was then opened and examined, for the purpose of discovering the seat and cause of the fatal effusion. On cutting away the hemisphere of the left side of the brain, a large semifluid clot was discovered, filling an apoplectic cell in the substance of the brain, just above the corpus striatum, and close to the corpus callosum: the size was that of a hen's egg. A little further examination discovered the source of hæmorrhage to be a large branch of the middle cerebral artery, the trunk of which vessel appeared more dense and white than usual, as if undergoing the change prior to ossification. The disease of which he died sufficiently attested the freedom of the circulation, as well as its force in the cerebral vessels, on the side on which the carotid had been tied. The arteries on the left side of the brain were rather larger than those on the opposite side. The anterior cerebral artery was of the same size as its fellow; the middle cerebral larger than that on the right side, which was filled with a coagulum, and did not admit the injection.* The large size of the latter vessel is accounted for by the increased size of the communicating branch; which, receiving its blood from the basilary, had become as large as an ordinary radial artery. The basilary appeared to be of its usual capacity, although it was evidently the channel which supplied the middle cerebral artery. The blood probably found an easier course from the basilary, through the left communicating branch, than into the right corresponding vessels, which appeared rather diminished in size. From an inspection of the base of the brain, after the vessels had been injected, it immediately struck the observer, that the left side of the arterial circle of Willis was much more developed than the right, and that the left side of the brain received its full share of arterial blood. The anterior cerebral artery received its supply from its fellow by means of the transverse branch: these vessels seemed to be of their usual size. The internal carotid

* These appearances are shewn in the "Reports," by a coloured lithographic diagram.

artery was pervious for about half an inch, and of its ordinary capacity.

The external vessels were not so well displayed. Those of the face did not receive the injection. The common carotid trunk was impervious throughout its whole extent, being reduced to a mere cord. The external carotid was injected at its commencement; and the superior thyroideal was also filled from the arteries of the opposite side: but beyond this the branches were empty, and therefore could not be satisfactorily traced.

It is rather to be regretted that Sir Astley was unable to determine with more precision the exact site of the aneurysm, and condition of the primary branches of the external carotid, as well as that of the internal carotid. The perviousness of the external carotid, at its origin, seems to shew that the aneurysm was, as Sir Astley thought at the period of the operation, situated in the internal carotid.

This case, taken with the one that precedes and follows it, must be deemed a valuable contribution to our information on the consequences of the operation for aneurysm. We know pretty well, for anatomical reasons, where to look for those anastomoses which shall stand in the stead of the obliterated or choked-up trunk; but still it is desirable to know what steps Nature has actually taken in these circumstances. The profession must feel indebted to Sir Astley for contributing these cases to the publication before us.

### III. Axillary Aneurysm cured, by tying the Subclavian Artery. Dissection of the Limb Twelve Years after the Operation.

This case, so far as the operation is concerned, was published in the 13th volume of the Med.-Chirurgical Transactions. The dissection of course had not then occurred; and, as this forms the principal interest of the details, to this we shall almost exclusively confine ourselves.

*Case.* A man, aged 36, while making a sudden exertion with his left arm in July, 1823, felt something snap below the collar-bone. In a day or two afterwards an aneurysmal tumor appeared below the clavicle. The operation of tying the subclavian artery was performed by Mr. Key on the 20th of September, the tumor being then of some size, and having thrown up the clavicle. The ligature separated from the artery on the 12th day after the operation. The tumor gradually subsided, and the patient lived until the Spring of 1835, when he died with anasarca of the lower limbs, and other symptoms of visceral disease. He had bequeathed his arm to Mr. Key, and after his death it was injected, removed from the body, dissected carefully, and put up as a preparation for the museum of the hospital. We may observe that the case possesses some additional interest, from its being the first instance in which the subclavian was tied with success in the hospitals of London.

*Dissection.* The muscles of the right arm were more than usually large. There was a small and indistinct cicatrix in the skin above the clavicle.

On removing the skin, the division of the platysma myoides was apparent, from an interspace of an inch between its fibres, which were here replaced by a dense cellular structure. The omohyoideus was rather higher than usual, probably from the division of the fascia which braces it down to the clavicle.

The subclavian trunk had undergone no material alteration in size, from its origin, to the point where the ligature had been applied, just on the outer edge of the scalenus muscle. Here the vessel became suddenly obliterated, assuming the form of a dense flattened cord, which was continued for about two inches and a half into the axilla, and terminated in the remains of the aneurysmal sac. The precise spot where the artery had been tied was clearly indicated by a deep indentation; but the continuity of the vessel above the ligature, with the obliterated portion below it, was not destroyed, or, more properly speaking, had been restored, after the separation of the silk, by a process of adhesion, which connected the two extremities to each other, and glued them to the contiguous structures. The aneurysmal sac still existed in the axilla; where it formed a firm and solid, but at the same time somewhat elastic and yielding tumor, about the size of a small hen's egg. It was situated immediately behind the pectoralis minor, and firmly adherent to the second rib; which, however, had not become, in the slightest degree, absorbed by the pressure which it must have sustained: the sac was otherwise but slightly connected to the surrounding textures, and presented a smooth uniform exterior, bearing altogether considerable resemblance to those cysts which are occasionally found to form themselves around foreign bodies. The obliterated portion of the axillary trunk terminated in the upper and back part of the sac; while, from its under surface, the continuation of the artery was seen to emerge, as a perfect vessel; having been restored to nearly its natural calibre by the entrance of a large branch, which was originally given off immediately below the tumor, and through which the blood had afterwards assumed a retrograde course.

On opening the sac, the coats of which were remarkably dense and hard, it was found to contain a firm and solid coagulum, which readily separated from the surrounding cyst, and, on being removed, retained the precise shape of the tumor. A section of it clearly evinced that it consisted of fibrin, apparently inorganized, dense, and tough in its texture, and of a dirty-yellowish colour.

The anastomosing vessels which carried on the circulation are too numerous to be individually described. They are, therefore, arranged into three sets:—

1. A posterior set, consisting of the supra-scapular and posterior-scapular branches of the subclavian, which anastomosed with the infra-scapular from the axillary.

2. An internal set, produced by the connexion of the internal mammary on the one hand, with the short and long thoracic arteries and the infra-scapular on the other.

3. A middle or axillary set, which consisted of a number of small vessels, derived from branches of the subclavian above, and passing through the axilla, to terminate either in the main trunk, or some of the branches of the axillary below. This last set presented most conspicuously the peculiar character of newly-formed, or rather of dilated arteries. They were excessively tortuous, and formed a complete plexus, which was almost inseparably connected with the axillary nerves: many of the branches penetrating into the midst of the nervous fibres, so as to render their separation a work of great difficulty and labour.

The chief agent in the restoration of the axillary trunk below the tumor was the infra-scapular artery, which communicated most freely with the internal mammary, supra-scapular, and posterior-scapular branches of the subclavian; from all which it received so great an influx of blood, as to dilate it to three times its natural size. The infra-scapular artery was, in this subject, given off much higher than usual; and its origin had been included in the aneurysmal dilatation: in fact, the artery opened into the sac itself; and, under the restored state of the circulation, the blood had to traverse a small portion of the cavity, in order to reach the commencement of the axillary trunk. The continuity between the two vessels had been preserved through the coagulum contained in the tumor; which, for a short space, actually constituted the arterial coats: thus, when the contents were removed, the injected wax became exposed at the bottom of the cyst; while a corresponding deep sulcus in the coagulum indicated the channel through which the blood had passed.

The supra-scapular artery was, in this instance, given off by the superficial cervical, and became augmented, just as it reached the scapula, by a branch which arose from the obliterated portion of the main trunk; but which had again been rendered available, as a medium of circulation, by receiving a vessel from the subclavian above.

The common origin of the short thoracic and humeral thoracic arteries had become obliterated as it came off from the sac itself: but the two vessels had subsequently regained their original size; the one being supplied by its connexion with the internal mammary; the other, by communications with the superficial cervical.

Mr. Key observes, in reference to the preceding case, that it tends to lessen our confidence in the dogma, that it is unsafe to apply a ligature to a large artery, close to the origin of a considerable branch. We would say, that this opinion, or this dogma, call it which we may, is founded upon facts too well attested to permit us to doubt, that the contiguity of the origin of a large arterial branch, is a source of risk. Such cases as the present may diminish our estimate of the quantum of that risk, but were they much more numerous than they are, they could only form a *quantitative* argument. The cases in which secondary hæmorrhage has occurred, when an artery has been tied close to the point from which a vessel of any size has sprung, are sufficient to make every prudent surgeon avoid, if possible, incurring such a hazard. On the other hand we have cases, which, like that before us, inform the surgeon that under circumstances otherwise favourable, he may hope that even the vicinity of a large branch will not prevent the adhesive process in the trunk, in the situation of the ligature. Probably the state of the patient's constitution exercises the most important influence. If that is good, the formation of a solid clot may be of comparatively little consequence; if that is bad, every collateral assistance is required to seal the mouth of the vessel. All surgeons know that the adhesive process occurs more readily in some persons than in others, even in what appears to be the state of perfect health. One man who, to use the popular expression, has "got good flesh to heal," repairs even the severest injuries with little effort and disturbance. In another, the most trifling wound will fester, the reparative process is established with difficulty, and imperfectly carried on, and severe lesions are almost surely mortal.

To revert to the subject of the preceding observations, the case we have related does not shake, and cannot shake the value of the practical rule—not to tie a large trunk near the source of an important branch, unless imperatively called upon to do so. If we have the choice of two situations, one near the origin of a considerable branch, and one remote from it, the man must be mad who would cæteris paribus select the former. But if we have no choice, or if there are serious objections to operating any where but near the origin of a large vessel, this case of Mr. Key's, and others of the same description, will tend to moderate our anxiety and apprehensions.

### IV. Femoral Aneurysm cured by tying the External Iliac Artery.

The facts of this case may be quickly summed up. The patient was a plethoric man, aged 26, admitted in September, 1822, with an aneurysm in the right femoral artery. The disease had commenced a year previously, when he also observed an aneurysm in the other groin; the latter, however, spontaneously disappeared. On the 28th of September Mr. Key tied the external iliac artery, in the manner recommended by Sir Astley Cooper. A single ligature only was employed. The only unpleasant symptoms that succeeded the operation, were headache and febrile disturbance. Purgatives and one bleeding to ten ounces removed them. The ligature was found lying loose in the wound on the 10th of October. The wound was itself quite healed on the 22nd. On the 25th, he could walk out of doors, and on the 28th he left the hospital in good health, with scarcely any remains of the aneurysmal sac.

Two cases of popliteal aneurysm, cured by the ligature of the femoral artery, are related at length by the Reporters. We need not pause to notice them.—Mr. Key appends to the series some general remarks on the subject of aneurysm, from which we shall select the following, on the removal of the ligature after time has been allowed for the adhesive process in the artery.—After having alluded to the circumstance, that attempts have been occasionally made to withdraw the ligature after a certain, that is, a very uncertain period, Mr. Key proceeds :—

"One of the earliest attempts of this kind, with which I am acquainted, was made by Sir Astley Cooper, in the year 1816, when I was dresser in the hospital. A miller, below forty years of age, had laboured for some months under pain, stiffness, and swelling under the knee, which had originally been produced by the act of lifting a heavy sack of flour. The tumor pulsated strongly, and occupied the whole popliteal space, being somewhat above the usual size of popliteal aneurysms. The man was otherwise healthy.—Sir Astley Cooper laid the artery bare, and passed around it a single stout silk ligature; securing it by a slip knot, so that it might be removed at any period the operator might choose. The ligature was allowed to remain undisturbed on the vessel for forty-eight hours; at the expiration of which time the lips of the wound were separated, the ligature exposed, and, by gentle means, loosened from the artery, but not removed. While Sir Astley Cooper was loosening the silk, I kept my hand on the sac, in order to ascertain if the adhesive process had sealed the artery, sufficiently to prevent the access of the blood. For a few seconds, the sac remained free from pulsation; but an impulse was soon felt, that rendered the re-application of the ligature necessary. The ligature was again tightened; and the pulsation in the swelling again ceased. The wound, being disturbed in the process of adhesion, suppurated. At the expiration of twenty-four hours after the second application of the ligature, making in all seventy-two hours from its first application, it was entirely removed, having completely sealed the artery, and effectually interrupted the current of the blood. The patient went on well for ten days; the wound granulated speedily; and the tumor remained free from pain, and diminished in size. On the evening of the eleventh day, a profuse hæmorrhage took place from the wound, but spontaneously ceased. I sat by his bedside during the night, in the expectation of a return of the bleeding; but no further loss of blood took place; and the wound, which for a few days bore an unhealthy aspect, re-assumed its former healthy state, and was healed at the end of seventeen days. He left the hospital in six weeks from the operation, able to walk well, and in good health."

At present we have not sufficient data to enable us to determine when the process of adhesion is sufficiently advanced, to permit the safe removal of the ligature. In the first place, that period probably differs in different persons, according to their constitutional powers; and, in the second place, the attempt to untie the knots is too likely to disturb the vessel, and derange the lymph effused in it. When we add to these circumstances the fact, that the present operation answers well, and that the accidents from the long retention of the ligature are neither numerous nor great, we shall account for the reluctance which surgeons have evinced to prosecute many such experiments as were made by Sir Astley Cooper. At the same time it is possible, that advantages might be obtained from an earlier abstraction of the ligature than is now effected.

Mr. Key mentions a suggestion which was made to him by a medical gentleman, with respect to the mode of undoing the knots of the ligature. It consists of thread passed under each fold of the knot, so that the ligature may be loosened by pulling the ends of the threads: catgut forms a good material for the ligature, as the knot more readily yields.

The cases we quit derive some degree of additional interest from the well known adroitness, and, we believe, success of Mr. Key, as an operator.

Here we must take our leave of the "Guy's Hospital Reports" for the present. Those reports contain the following articles:—An Introduction on the Advantage of Recorded Experience in Medicine, by Dr. Barlow—Observations on Fever, by Dr. Bright—Unusual Dislocation of the Head of the Femur upwards on the Ilium—Cases of Dislocation and Fracture, with Observations, by Mr. Bransby Cooper—Tetanus, cured by quinine and stimulants—Case of Traumatic Tetanus—Cases in the Obstetric Ward—Cases of Small-pox in the Deaf and Dumb Asylum—Illustrations of the Museum.—These papers we have not yet had space to notice. The remaining contents of the volume have been fully analysed in our present clinical department. They are the following:—Effects produced by Diseased Cerebral Arteries, by Dr. Bright—Ovarian Dropsy removed by the Accidental Rupture of the Cyst—Cases of Femoral, Carotid, Axillary, and Popliteal Aneurism—Case of Hydrocele of the Neck—Essay on Pericarditis, by Dr. Hughes.

Thus a large part of the contents of the Guy's and St. Thomas's Hospital Reports will be found in this number of our Journal. We shall endeavour to render our clinical department more and more complete as opportunities present themselves. This is essentially a practical Journal of Medicine and Surgery. We disclaim and reject the mysteries of transcendentalism, those emasculated fancies the offspring of the stagnant vapours of the closet, and food only for pedantic stomachs. Facts and strict inductions on them, the pabulum and exercise of manly intellect, are what we mainly profess to offer.

---

### Dreadnought Hospital Ship.

#### Supposed Aneurysmal Tumor in the Orbit, for which the Common Carotid Artery was tied.*

The following is in many respects so curious a case, that we lay an abstract of it before our readers.

*Case.* A seaman, aged 20, was admitted into the hospital ship on the 13th of July, 1835, with concussion of the brain, considerable hæmorrhage from the right ear, and a small wound behind the left. These were the consequences of a blow from the gaff of his vessel. On the 15th, there was complete deafness of the right ear—the integuments around the *left* orbit were œdematous—the pupil of that eye was dilated—he could not move the eye—and there was slight paralysis of that side of the face. On the 18th, there was some irregularity of the pupil, with headach on the left side, and some inflammatory symptoms. He was salivated. On the 24th, there was purulent discharge from the right ear. On the 28th, the conjunctiva of the left eye was much inflamed and œdematous. On the 31st, purulent matter was deposited between the laminæ of the cornea.

In the beginning of September, the anterior laminæ of the cornea ulcerated. In the middle of November he was seized with small-pox, and, after recovering from this in December, the eye was much inflamed, and the ulcer on the cornea large. The ulcer gradually filled up, yet the eye seemed to protrude.

"On the 1st of February," says Mr. Busk, the surgeon and the reporter of the case, "on making examination of the eye, I felt on pressing the globe a distinct pulsation; and farther found, deeply situated in the upper and inner part of the orbit, a firm pulsating tumor, which appeared about half an inch in its transverse and longest diameter: it was situated between the levator of the eyelid and the bone, and did not show itself externally, but when the eyelid was raised it caused some projection of the loose conjunctiva.

The pulsation of the tumor was accompanied with a very distinct whirr, which could also be felt on pressing the parts in its immediate neighbourhood. Through the stethoscope, a very loud aneurysmal whizzing sound was communicated, which could also be heard on applying the instrument over the inner canthus of the other eye, and on the left side of the frontal bone, as high as the roots of the hair, and nearly as far back as the ear."

In addition, there were loud noises experienced in the head. Pressure on the left common carotid artery arrested

---

* Med. Gazette, Feb. 27th, 1836.

the pulsation and sounds of the tumor, as well as the noises in the head.—Mr. B., therefore, determined to tie that artery, which he did on the 2d of February, after the abstraction of 20 ounces of blood on the 1st. Immediately on tightening the ligature, the pulsation and sounds of the tumor, and the noises in the head were stopped. Four hours after the operation, obscure pulsation could be felt in the tumor, and the whizzing sound could be heard in it, although there was no pulsation in the temporal; the sounds in the interior of the cranium were heard at intervals. On the 3d, he was bled to 16 ounces, on account of cough. On the 4th, no tumor could be felt in the orbit, and the pulse and the whizzing were gone. They did not afterwards recur, at least they had not done so by the 19th, the date of the report. On the 15th, the ligature separated from the carotid. The eye had then returned nearly to its natural level. The upper half of the cornea was clear, the lower occupied by a dense leucoma; there was no power of motion in the globe of the eye, nor in the left side of the face; there was great deafness, especially in the left ear. We would state that on the 6th some grumous blood was discharged from the left nostril. Mr. Busk promises to communicate hereafter any particulars of consequence, with which he may become acquainted.

The case is curious, and we cannot offer a definitive opinion on it. Supposing the tumor to have been aneurysmal, and to have been seated in one of the branches of the ophthalmic artery, it is singular that it should so suddenly disappear after tying the common carotid. The communication of one internal carotid with the other in the cranium is so free, that this effect of the ligature is remarkable. Speculation on the case, however, is useless, and we must content ourselves with simply recording the fact.

---

### St. George's Hospital.

### Sir Benjamin Brodie on the Treatment of Corns and Bunions.

Sir Benjamin Brodie has not deemed it derogatory to the majesty of surgery, to look after corns and bunions. From a most excellent clinical lecture on this subject by the able Baronet, published in the Medical Gazette, we extract some practical remarks upon their management.

A corn at first consists merely of a thickening of the cuticle, the effect of pressure, and usually seated over some projecting bone or articulation. But after a time a little bursa forms beneath it, so that a perfect corn consists of both conditions. If the corn occurs between the toes it is kept moist, and constitutes a "soft corn."

Hard corns are temporarily relieved by paring, filing, or causing them to exfoliate by means of caustics. But more is required for a radical cure. The following are the remarks of Sir Benjamin on this head.

" In some way or other all undue pressure must be removed from the part on which the corn is situated.—*First*, the shoe must be made as nearly as possible to the shape of the foot, and it must cover the metatarsis and a portion of the tarsus, so that the whole pressure may not be thrown on the toes; or a boot made to be laced or buttoned may be worn instead of a shoe. In some cases it is advisable that the shoe or boot should be made, not of ordinary leather, but of very soft and flexible buckskin, or of cloth. A material for shoes and boots is sold under the grandiloquent name of *pannus corium*, which answers the purpose intended in these cases very well. It is really a kind of cloth, but it has the appearance of leather, and is very soft and pliable. *Secondly*, if any of the toes are displaced in any of the ways which I have before described, we must endeavour to restore them to their natural position. In young persons this may be generally accomplished. A contrivance made use of by the bandage-makers is very useful on these occasions. It consists of a thin plate of metal covered with thin leather, or a piece of strong leather, fitted to the lower surface of the foot, —not to the whole of the surface, but extending from the extremities of the toes nearly to the tarsus. Slits are formed in this plate of metal or leather, and tapes are passed through these slits, forming loops above, by means of which the toes are bound down and retained in their proper places. In many cases the same object may be attained by simpler means. A stripe of linen, spread with adhesive plaster, about two-thirds of an inch in breadth, may be passed over the toes which are too elevated, and under the others, the extremities of the plaister being made to cross each other over the metatarsus. If this be neatly applied, it will keep the toes parallel to, and on the same level with, each other. Whichever of these methods be employed, it is necessary that it should be persevered in for a considerable time."

In old people it is useless to attempt to alter the malposition of the toes. Sometimes it is necessary to amputate a toe, in consequenee of the inconvenience it occasions. This necessity arises more frequently in the lower than in the upper classes.

The chiropodists relieve a corn, by cutting a hole corresponding to it in a piece of buckskin leather, spread with some adhesive plaister, and applying this to the foot. Of course the pressure is thus taken off the corn. If a hard corn forms on the sole of the foot, over the head of one of the metatarsal bones, it is particularly troublesome. Sir Benjamin thus treats it. The hard cuticle being removed, a broad piece of buck-skin leather is to be applied, having a hole in it where the corn is situated. But a thin piece of calico spread with adhesive plaster, and having no hole in it, is to be applied first; that is, between the leather and the foot. Without this last contrivance the flesh of the foot, when the patient walks, bulges or projects into the hole of the leather, so as completely to fill it up, and the patient's condition is rendered rather worse than better. The calico with adhesive plaster prevents this inconvenience, at the same time that it does not prevent the leather answering the intended purpose of taking the pressure off the corn, and throwing it on the surrounding parts.

When an abscess forms, as it occasionally does, in the bursa under a corn, the corn should be pared and the bursa opened.

A soft corn is to be treated on the same principle as a hard one. Pressure is to be removed. A properly shaped shoe must be made, and the toes if displaced brought back by appropriate contrivances to their right position. The little toe is the one usually distorted, and it is pushed more or less underneath the second phalanx of its neighbour. The following contrivance sometimes answers:—A piece of *very thick* buck-skin leather, spread with adhesive plaster, is to be applied on the inside of the little toe, so as to occupy the whole of the inner surface, from the apex to the second joint. The leather should be cut so as to be thin at its margin; and it should be sufficiently broad to admit of being doubled over a good part of the upper and under surface of the toe, as well as its extremity. This contrivance will keep the little toe at some distance from the next toe, and prevent it from sliding again under it. In addition to these means the nitric acid should be applied to the corn itself, so that it may dry, and exfoliate. Occasionally a soft corn is exquisitely painful, although there is no suppuration beneath it. In a case which occurred to Sir B. Brodie the application of the nitric acid relieved the excessive tenderness.

The treatment of bunions is regulated by much the same principle as that of corns.

Unhappily we can speak from experience on the miseries of corns. The tendency to them is certainly frequently hereditary, the children of parents who have laboured under them, contracting them long before adequate compression from boots has occurred to occasion them. When a great disposition to their formation exists, we much fear that a permanent cure is next to an impossibility. We have given up the attempt in despair. We have tried, ad nauseam, cut boots, cut corns, plaisters of all sorts on leathers of all thickness, caustics, and all the resources of chiropodism, and here we are hobbling in damp weather, with unabated agonies. If a man has really such corns, we pity him. Let him find, if he can, an honest bootmaker, who will make him what it is as difficult to discover, as the longitude, easy boots, and patiently suffer the inflictions of Providence. That is the conclusion to which we have been compelled to come.

But to those who are not thus doomed to hopeless torment, we recommend the capital lecture of Sir Benjamin. If the remedies he proposes won't ease their feet, they may join the awkward squad of incurables, for neither chiropodism nor surgery can do more for them.

